# Spotlight influenza: Estimation of influenza vaccine effectiveness in elderly people with assessment of residual confounding by negative control outcomes, Finland, 2012/13 to 2019/20

**DOI:** 10.2807/1560-7917.ES.2021.26.36.2100054

**Published:** 2021-09-09

**Authors:** Ulrike Baum, Sangita Kulathinal, Kari Auranen

**Affiliations:** 1 Department of Health Security, Finnish Institute for Health and Welfare, Helsinki, Finland; 2 Department of Mathematics and Statistics, University of Helsinki, Helsinki, Finland; 3 Department of Mathematics and Statistics, University of Turku, Turku, Finland; 4 Department of Clinical Medicine, University of Turku, Turku, Finland

**Keywords:** bias, cohort studies, confounding factors, influenza, negative control outcomes, vaccine effectiveness

## Abstract

**Background:**

Cohort studies on vaccine effectiveness are prone to confounding bias if the distribution of risk factors is unbalanced between vaccinated and unvaccinated study subjects.

**Aim:**

We aimed to estimate influenza vaccine effectiveness in the elderly population in Finland by controlling for a sufficient set of confounders based on routinely available register data.

**Methods:**

For each of the eight consecutive influenza seasons from 2012/13 through 2019/20, we conducted a cohort study comparing the hazards of laboratory-confirmed influenza in vaccinated and unvaccinated people aged 65–100 years using individual-level medical and demographic data. Vaccine effectiveness was estimated as 1 minus the hazard ratio adjusted for the confounders age, sex, vaccination history, nights hospitalised in the past and presence of underlying chronic conditions. To assess the adequacy of the selected set of confounders, we estimated hazard ratios of off-season hospitalisation for acute respiratory infection as a negative control outcome.

**Results:**

Each analysed cohort comprised around 1 million subjects, of whom 37% to 49% were vaccinated. Vaccine effectiveness against laboratory-confirmed influenza ranged from 16% (95% confidence interval (CI): 12–19) to 48% (95% CI: 41–54). More than 80% of the laboratory-confirmed cases were hospitalised. The adjusted off-season hazard ratio estimates varied between 1.00 (95% CI: 0.94–1.05) and 1.08 (95% CI: 1.01–1.15), indicating that residual confounding was absent or negligible.

**Conclusion:**

Seasonal influenza vaccination reduces the hazard of severe influenza disease in vaccinated elderly people. Data about age, sex, vaccination history and utilisation of hospital care proved sufficient to control confounding.

## Introduction

In Finland, influenza vaccination for those 65 years and older was added to the vaccination programme in 2002 and is available free of charge in public health centres. The Finnish Vaccination Register, established 10 years later, records all vaccinations administered in the programme since 2009 [1]. The vaccines are selected by the Ministry of Social Affairs and Health in open tendering processes. Trivalent inactivated influenza vaccine was used until the end of season 2017/18 and thereafter replaced by a tetravalent inactivated influenza vaccine.

Assuming that influenza vaccination provides partial protection for the vaccinated, an appropriate measure of the protective direct effect under real-life conditions is influenza vaccine effectiveness (IVE) defined as the vaccine-attributable relative reduction in the hazard of influenza disease [2,3]. To guide public health measures as well as individual-level decision-making, IVE in elderly people is estimated regularly in Finland using a register-based cohort study design [4].

Observational studies estimating IVE are prone to confounding caused by an unbalanced distribution of risk factors between the vaccinated and unvaccinated. If such imbalance is not controlled, IVE may be underestimated (confounding by indication) or overestimated (healthy vaccinee bias) [5]. This raises the question about which factors confound the true association between vaccination and influenza disease and how to balance them adequately.

Numerous chronic diseases, e.g. chronic pulmonary disease or diabetes mellitus, qualify as potential confounders since chronically ill people have a higher risk for influenza [6] and choose more often to be vaccinated than healthy people [7]. Another potential confounder is influenza vaccination history [8]. Firstly, the influenza risk of previously vaccinated individuals may be affected by a lower risk of infection in the past or residual vaccine-induced immunity. Secondly, the health-seeking behaviour of those previously vaccinated makes them more likely to be vaccinated again. Other factors frequently considered in studies of IVE in elderly people are age, sex, healthcare utilisation, medications, nursing home residency, socioeconomic status and smoking status [5,9]. A standard method to control for confounding is adjustment for a set of covariates.

Here, the objective was to obtain confounder-adjusted estimates of IVE in the elderly population in Finland based on medical and demographic register data. To achieve the objective, we asked whether routinely available individual-level data about age, sex, vaccination history and utilisation of hospital care sufficiently capture relevant confounding patterns in the population. Using off-season hospitalisation for acute respiratory infection (ARI) as a negative control outcome, we assessed the residual confounding bias remaining after covariate adjustment.

## Methods

### Data sources

The Population Information System and the Finnish Vaccination Register provided demographic and vaccination data, respectively. The Care Register for Health Care and the Finnish Infectious Diseases Register provided diagnostic information and laboratory-confirmed data. A detailed description of these data sources and linkage can be found elsewhere [4].

### Study periods

We assessed the timeline of influenza virus circulation in Finland by monthly numbers of influenza-positive laboratory tests from August 2012 through May 2020 and defined October to May as influenza season, June as transitional period and July to September as influenza off-season. The study periods were the eight influenza seasons 2012/13 to 2019/20 and the seven influenza off-seasons 2013 to 2019.

### Estimation of vaccine effectiveness

For each season, we conducted a register-based cohort study to estimate IVE [4]. The study cohort consisted of the population aged 65–100 years (see Supplementary Table S1 for the inclusion/exclusion criteria). The outcome was laboratory-confirmed influenza, further identified as influenza A or B. The exposure was seasonal influenza vaccination including pre-seasonal vaccinations given in August or September. Three time-dependent exposure states were distinguished: unvaccinated, vaccinated less than or exactly 14 days ago, and vaccinated more than 14 days ago (fully vaccinated).

The covariates used as confounders were age (65–69; 70–74; 75–79; 80–84; 85–100 years), sex, vaccination status at the end of the previous season, nights hospitalised in the 5 years before season onset, and presence of underlying chronic conditions defined based on the subject-specific 1-year history of inpatient and outpatient hospital visits before season onset (see Supplementary Table S2 for the ICD-10 diagnostic codes [10]).

Each study subject was considered at risk for the outcome (influenza of any type, influenza A, or influenza B) from season onset until the first of the following events: outcome, death or end of study. The IVE was quantified as 1 minus the covariate-adjusted hazard ratio (HR). Using Cox regression with time since season onset as the underlying time scale, we estimated crude and covariate-adjusted HR comparing the hazard in the fully vaccinated with the hazard in the unvaccinated. The validity of the proportional hazards assumption was examined visually by plotting nonparametric estimates of HR over time [11].

As a sensitivity analysis, we investigated to what extent the outcome’s specificity influenced the IVE estimates. To this end, we replaced the highly specific outcome of laboratory-confirmed influenza with hospitalisation for ARI as a related but unspecific outcome, expecting to observe smaller IVE levels than in the main analysis. We defined hospitalisation for ARI as any inpatient hospitalisation or emergency room visit for acute upper respiratory infections (ICD-10 diagnostic codes J00–J06), pneumonia (J12–J18), other acute lower respiratory infections (J20–J22), chronic obstructive pulmonary disease with acute lower respiratory infection (J44.0), cough (R05) or unspecified fever (R50.9). Study subjects already hospitalised for ARI at season onset were excluded. As hospital data were only available until the end of 2019, this sensitivity analysis was limited to the influenza seasons 2012/13 to 2018/19.

### Detection of residual confounding

To detect residual confounding, we measured the association between a negative control outcome and influenza vaccination (see Supplementary Table S3 for the inclusion/exclusion criteria). By definition, a negative control outcome must not be affected by the exposure but, at the same time, it must be affected by the same factors that influence both the actual outcome and exposure [12]. Based on this definition, we here chose off-season hospitalisation for ARI as the negative control outcome and conducted a register-based cohort study for each influenza off-season. The exposure was receipt of at least one influenza vaccination in the preceding season. The covariates were the same as those used to estimate IVE.

Each study subject was considered at risk for the negative control outcome from off-season onset until the first of the following events: negative control outcome, off-season influenza vaccination, death or end of off-season. When the 95% confidence interval (CI) for the crude HR did not include the value of 1, we inferred that there was a spurious association between the negative control outcome and vaccination, indicating confounding. When the 95% CI for the covariate-adjusted HR did include the value of 1, residual confounding was deemed unlikely.

To further investigate the adequacy of the selected covariates, we defined a large number of alternative covariate sets by refining covariate definitions and including two-way covariate interactions. The refined covariate definitions were (i) age classified into nine categories allowing for more gradual changes in frailty, vaccine uptake and vaccination history among the younger age groups, (ii) influenza vaccinations received in the previous three seasons, (iii) nights hospitalised in the 3 years or in the year before season onset and (iv) presence of underlying chronic conditions based on the 3- or 5-year history of hospital visits before the season preceding the off-season in question. A total of 655 covariate sets were used to adjust the HR for confounding when comparing the hazards of the negative control outcome in the vaccinated and unvaccinated. The sum of the absolute values of the logarithms of the HR from the seven off-seasons was calculated for each of the 655 sets. In the absence of confounding, the expected value of each sum equals 0. The 655 sets were ranked by the sum to identify the sets performing best across all seven off-seasons (Supplementary Figure S1).

All analyses were performed in R 3.6.3 (R Foundation for Statistical Computing, Vienna, Austria).

### Ethical statement

The study was approved by the institutional review board of the Finnish Institute for Health and Welfare (THL/607/6.02.00/2016).

## Results

**Figure 1:**
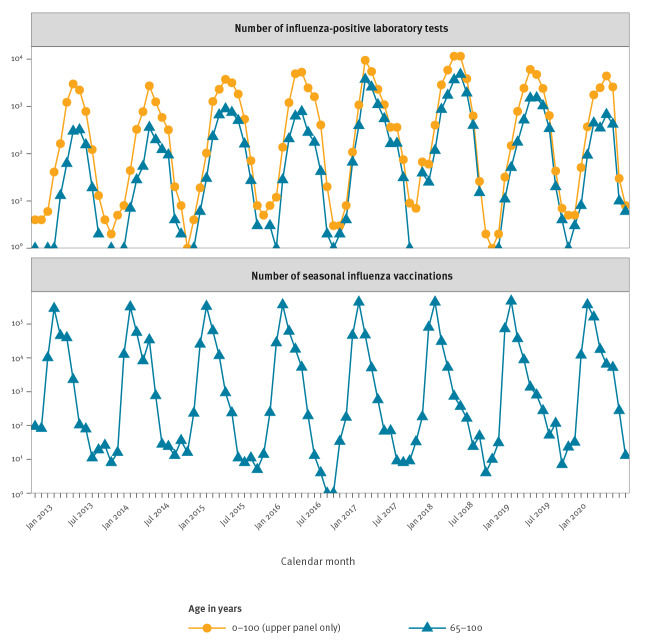
Timeline of influenza virus circulation and seasonal influenza vaccination, Finland, August 2012–May 2020

Figure 1 shows the timeline of influenza virus circulation and vaccination from August 2012 through May 2020. Although the epidemics clearly ceased during the off-seasons, sporadic respiratory specimens were tested influenza-positive even then. In most seasons, the vaccination campaign was launched 2–3 months before the epidemic peak.

### Study cohorts and vaccine uptake

The sizes of the study cohorts varied between 803,653 (2012/13) and 1,125,913 (2018/19) individuals (Table 1). Each season, more than 70% of the population aged 65–100 years were included (Supplementary Table S1). The vaccination coverage varied between 37% (2012/13) and 49% (2018/19) and was highest in the subgroup of previously vaccinated individuals (Supplementary Table S4). The median vaccination date in this subgroup was 1–6 days earlier than in those not vaccinated previously. In addition, vaccinated individuals were more likely to be chronically ill or hospitalised in the past than unvaccinated individuals (Supplementary Table S4). The administered vaccine brands are listed in Supplementary Table S5.

**Table 1:** Influenza vaccine effectiveness against laboratory-confirmed influenza in people aged 65–100 years, Finland, August 2012–May 2020

Season	Cohort size	Unvaccinated		Fully vaccinated		Vaccine effectiveness^a^ %	
		Cases	Attack rate^b^	Cases	Attack rate^b^	Estimate	95% CI
2012/13	803,653	441	89	185	64	31	15–44
2013/14	830,872	470	97	205	63	45	32–56
2014/15	951,858	1,770	316	1027	276	22	14–29
2015/16	991,932	1,357	242	495	129	48	41–54
2016/17	1,024,822	4,670	856	3,006	672	24	19–28
2017/18	1,069,897	6,650	1,205	5,263	1,053	16	12–19
2018/19	1,125,913	2,690	480	2,088	383	26	20–31
2019/20	1,107,226	1,095	182	679	149	24	15–33

CI: confidence interval.

^a^ Vaccine effectiveness was defined as 1 minus the covariate-adjusted hazard ratio. The covariates were age, sex, one-year vaccination history, nights hospitalised in the past 5 years and presence of underlying chronic conditions.

^b^ The attack rate is presented as the cumulative risk at the end of the study period multiplied by 10^5^.

### Vaccine effectiveness against laboratory-confirmed influenza

The smallest and highest numbers of influenza cases were observed in 2012/13 and 2017/18, respectively (Table 1). From 2012/13 through 2017/18, more than 80% of the laboratory-confirmed cases were hospitalised within 7 days after the influenza-positive specimen was sampled (Supplementary Table S6). The proportions of hospitalised cases were similar among vaccinated and unvaccinated cases.

Visual examination of the time dependency of the covariate-adjusted HR supported the proportional hazards assumption. After initial fluctuations, the HR estimates converged around the epidemic peak (Supplementary Figure S2).

IVE against laboratory-confirmed influenza ranged from 16% (95% CI: 12–19) in 2017/18 to 48% (95% CI: 41–54) in 2015/16 (Table 1). The effect of covariate adjustment was heterogeneous (Supplementary Table S7). Without adjustment, IVE would have been underestimated by 10 percentage points in 2013/14 and 2014/15 but was basically unchanged in 2012/13 and 2015/16.

**Figure 2:**
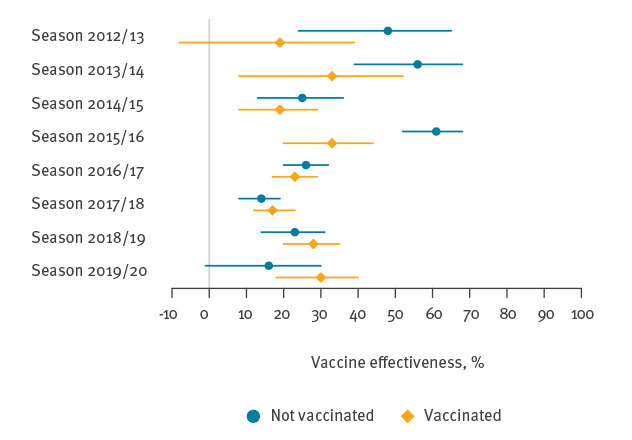
Influenza vaccine effectiveness against laboratory-confirmed influenza in people aged 65–100 years, stratified by vaccination status at the end of the previous season, Finland, August 2012–May 2020

Figure 2 and Supplementary Table S8 show the IVE estimates stratified by vaccination status at the end of the previous season. In the earlier seasons IVE in previously vaccinated individuals was lower than IVE in individuals who had not been previously vaccinated. This trend, however, disappeared or was inverted in the more recent seasons.

Data points: point estimates; lines: 95% confidence interval estimates. The size of each cohort is given in Table 1 and the size of each stratum is given in Supplementary Table S8.

**Figure 3:**
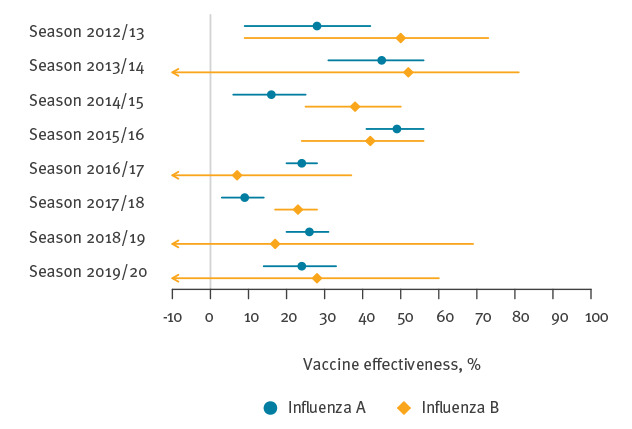
Influenza vaccine effectiveness against laboratory-confirmed influenza A and B in people aged 65–100 years, Finland, August 2012–May 2020

Figure 3 and Supplementary Table S9 show the IVE estimates by virus type. In all eight seasons, the majority of cases were caused by influenza A. Consequently, IVE against influenza A was (i) similar to the overall IVE as presented in Table 1 and (ii) estimated with higher precision than IVE against influenza B. In the two seasons for which the precision allowed meaningful comparisons of virus type-specific IVE (2014/15 and 2017/18), vaccination provided stronger protection against influenza B than against influenza A.

Data points: point estimates; lines: 95% confidence interval estimates. The size of each cohort is given in Table 1.

### Vaccine effectiveness against acute respiratory infection

The smallest and highest numbers of cases hospitalised with ARI were observed in 2013/14 and 2017/18, respectively (Table 2). In six of seven seasons considered in this sensitivity analysis, the attack rate in fully vaccinated individuals was higher than the attack rate in unvaccinated individuals (Table 2). Covariate adjustment, however, corrected for the unbalanced distribution of risk factors and lowered the HR estimates (Supplementary Table S10, Supplementary Figure S3). The corresponding IVE estimates ranged from –2% (95% CI: –6 to 1) in 2018/19 to 6% (95% CI: 2–10) in 2013/14 (Table 2).

**Table 2:** Influenza vaccine effectiveness against hospitalisation for acute respiratory infection in people aged 65–100 years, Finland, August 2012–May 2020

Season	Cohort size	Unvaccinated		Fully vaccinated		Vaccine effectiveness ^a^ %	
		Cases	Attack rate^b^	Cases	Attack rate^b^	Estimate	95% CI
2012/13	802,597	13,822	2,439	6,717	2,799	−1	−5 to 2
2013/14	829,885	12,605	2,249	6,675	3,206	6	2 to 10
2014/15	950,866	16,594	2,627	8,818	4,674	4	1 to 7
2015/16	991,056	16,998	2,660	8,800	4,118	2	−1 to 5
2016/17	1,023,992	17,411	2,843	11,282	3,133	0	−3 to 3
2017/18	1,069,160	17,710	2,788	11,716	2,865	3	0 to 6
2018/19	1,125,151	14,584	2,150	9,457	2,057	−2	−6 to 1

CI: confidence interval.

^a^ Vaccine effectiveness was defined as 1 minus the covariate-adjusted hazard ratio. The covariates were age, sex, 1-year vaccination history, nights hospitalised in the past 5 years and presence of underlying chronic conditions.

^b^ The attack rate is presented as the cumulative risk at the end of the study period multiplied by 10^5^.

### Residual confounding

The sizes of the study cohorts in the seven off-season analyses varied between 770,550 individuals in 2013 and 1,089,843 in 2019 (Table 3, Supplementary Table S11). The attack rates were consistently higher in the vaccinated than in the unvaccinated (Table 3). Consequently, the crude HR were greater than 1. As none of the 95% CI included 1, the crude estimates were deemed to be confounded.

**Table 3:** Hazard ratios comparing the hazards of off-season hospitalisation for acute respiratory infection in vaccinated and unvaccinated people aged 65–100 years, Finland, August 2012–May 2020

Off-season	Cohort size	Unvaccinated		Vaccinated		Crude HR		Adjusted HR^b^	
		Cases	Attack rate^a^	Cases	Attack rate^a^	Estimate	95% CI	Estimate	95% CI
2013	770,550	3,278	680	2,695	947	1.40	1.33–1.47	1.08	1.01–1.15
2014	799,926	3,405	736	3,112	932	1.27	1.21–1.33	1.00	0.94–1.07
2015	917,978	3,995	737	3,409	917	1.25	1.19–1.30	1.01	0.96–1.07
2016	957,252	4,347	811	,3634	872	1.08	1.03–1.12	1.00	0.94–1.05
2017	989,040	4,063	793	4,050	857	1.08	1.03–1.13	1.00	0.94–1.05
2018	1,033,957	4,164	779	4,266	862	1.11	1.06–1.16	1.01	0.95–1.06
2019	1,089,843	2,971	547	3,408	628	1.15	1.09–1.21	1.07	1.01–1.14

CI: confidence interval; HR: hazard ratio.

^a^ The attack rate is presented as the cumulative risk at the end of the off-season multiplied by 10^5^.

^b^ The hazard ratio was adjusted for age, sex, 1-year vaccination history, nights hospitalised in the past 5 years and presence of underlying chronic conditions.

Adjustment for the five covariates for which IVE estimation was adjusted lowered the HR estimates and resulted in 95% CI that included the value of 1 in all but two off-seasons (Table 3). One of the two exceptional off-seasons (2013) exhibited the study’s greatest crude HR (1.40; 95% CI: 1.33–1.47). The adjusted HR was then estimated at 1.08 (95% CI: 1.01–1.15) suggesting that most confounding was actually controlled for. The hazards in vaccinated and unvaccinated individuals were proportional throughout all off-seasons (Supplementary Figure S4).

In the analysis of the 655 alternative covariate sets, 18 sets performed slightly better in controlling confounding than the set of five covariates used above (Supplementary Table S12). This set (rank 19) and the best set (rank 1) differed in how age was categorised and whether two-way interactions between age or presence of underlying chronic conditions and vaccination in the previous season were included. None of the top 40 sets contained the covariate ‘influenza vaccinations received in the previous three seasons’.

## Discussion

Based on individual-level medical and demographic data in the routinely available Finnish registers, we assessed IVE in elderly people in eight consecutive seasons. Because the majority of laboratory-confirmed cases required hospitalisation, the estimates pertain to a severe and highly specific outcome. In every season, vaccination reduced the hazard of severe influenza disease in those vaccinated by 16% to 48% but provided no or minimal protection against the unspecific outcome of hospitalisation for ARI. As the vaccinated were generally more frail, confounding by indication outweighed any healthy vaccinee bias. Residual confounding was deemed absent or negligible after covariate adjustment for only five covariates: age, sex, 1-year vaccination history, nights hospitalised in the past 5 years and presence of underlying chronic conditions.

In general, the estimated IVE levels are in line with other studies [13-17]. In particular, all these studies indicate that IVE in elderly people usually does not exceed 50%. In our study, IVE was highest (45% and 48%) in 2013/14 and 2015/16, seasons dominated by influenza A(H1N1) viruses (see Supplementary Table S13 for the distribution of influenza subtypes/lineages in Finland based on sentinel surveillance data). In the subsequent seasons 2014/15 and 2016/17, dominated by influenza A(H3N2) viruses typically associated with lower IVE and increased disease burden [13-15,18], IVE was reduced to half the level observed in the respective preceding season (22% and 24%). In 2017/18, a severe season characterised by co-circulation of influenza A(H3N2) and B/Yamagata viruses, IVE was at its lowest level (16%). Our results and those of a European multi-centre study reveal that the vaccines used in 2017/18 provided better protection against influenza B than against influenza A [16]. Since those vaccines did not contain virus of the Yamagata lineage, this suggests some degree of cross-lineage protection.

In contrast to a hospital-based study, which estimated the interim 2019/20 IVE in elderly people at a remarkably high value of 60% (95% CI: 39–74) [19], our 2019/20 estimate of 23% shows that IVE was actually not much different from that in previous seasons. We found, however, that the estimated HR was fluctuating before the epidemic peak, so that also our study would have resulted in a higher estimate had we reported interim IVE (see Supplementary Figure S2 for the estimated HR fluctuations). It remains unclear whether the observed decreasing trend was due to waning immunity, to a change in circulating strains or simply to the small initial number of cases. In general, there was no prominent pattern in how the HR behaved over different seasons. We thus infer that time since vaccination does not have a strong effect on IVE.

Because residual immunity may enhance or mitigate the immune response to vaccination, history of influenza vaccination may act as an effect modifier [20]. Stratification by previous vaccination status, however, revealed no clear trend across seasons, neither in the present study nor in another European study [21]. The vaccination dates in the two subgroups of previously vaccinated and not previously vaccinated were similar, ruling out the impact of time since vaccination on this comparison. As the stratified IVE estimates were greater than 0% across all seasons, our analysis demonstrates that both subgroups ultimately benefit from seasonal influenza vaccination.

To assess the adequacy of the selected covariates, we performed an in-depth analysis of residual confounding using a negative control outcome. As the negative control outcome, we chose off-season (i.e. post-season) hospitalisation for ARI, an unspecific outcome similar to laboratory-confirmed influenza in clinical picture and severity. While presumably unaffected by the exposure (no influenza circulation during off-seasons), this outcome should be affected by the same factors that influence both the actual outcome and vaccination. However, using such a post-season outcome as the negative control bears a risk of underestimating the impact of confounding. This is because the imbalance in frailty between the vaccinated and unvaccinated may diminish over time owing to the loss of frail individuals [22]. Therefore, we also estimated the HR of hospitalisation for ARI during the seasons and found that the HR was constant over time in most seasons. Consequently, it seems unlikely that any time trends would have led to underestimation of confounding bias in the off-season analyses. To confirm our findings, future research may consider additional (in-season and off-season) negative control outcomes.

The registers routinely available for Finnish IVE studies do not contain information on the study subjects’ medication, socioeconomic characteristics or lifestyle. Nevertheless, relying only on data about age, sex, vaccination history and utilisation of hospital care, we ascertained a set of factors that satisfactorily explained differences in the baseline risks between vaccinated and unvaccinated individuals. It therefore seems plausible that socioeconomic characteristics and lifestyle do not confound IVE estimation. This conjecture is in line with a Danish survey that found no association between income, education or current smoking and influenza vaccination in elderly people [7]. Interestingly, covariate sets that included both ‘nights hospitalised in the past’ and ‘presence of underlying chronic conditions’ performed better in the control of confounding than sets that included none or only one of the two (see Supplementary Table S12). This demonstrates that both factors were needed to address confounding by differential healthcare utilisation and frailty.

Covariate adjustment is a standard method to control for confounding but the number of covariates that can be considered in a multivariate analysis is usually limited by the size of the study. As an alternative method, recent IVE studies have therefore used the propensity score [23,24]. It summarises the conditional probability of vaccination given a large number of covariates and enables an automated selection of confounders in register-based studies [25]. However, propensity score methods are not necessarily superior to covariate adjustment and manual confounder selection based on subject matter knowledge [26,27]. We therefore chose the latter, simpler approach and minimised the number of covariates by defining a binary summary measure (presence/absence of any underlying chronic condition) instead of considering single conditions as confounders. When we found no major indication of residual confounding after adjusting for just five covariates, we saw no need to apply alternative methods to control for confounding.

If the adjusted HR of a negative control outcome has the expected value of 1 in the absence of other sources of bias, a study can be deemed free from residual confounding. In practice, however, studies are always prone to estimation error and residual confounding can thus never be ruled out with certainty. Our study is no exception. Nevertheless, we chose an explicit rule to infer adequate control of confounding when the 95% CI included the value of 1. In contrast to other studies [25,28] that draw conclusions about the absence/presence of residual confounding using negative control outcomes but do not specify rules for such decisions, we here aimed at transparency in our decision-making.

Apart from confounding, information bias may affect register-based IVE estimation. We addressed the problem of exposure measurement error due to missing vaccination data by excluding individuals living abroad or in municipalities incompletely covered in the Finnish Vaccination Register (see Supplementary Tables S1 and S3). By contrast, we had no means to control for outcome measurement error as the registers do not contain data about negative test results. All study subjects without a laboratory-confirmed influenza record were considered being at risk of severe influenza disease until the end of the season, although a mild infection could have immunised them without triggering hospitalisation and timely laboratory testing. Assuming that vaccination provides partial protection for all vaccinated, keeping those unidentified influenza cases in the risk set leads, over time, to underestimation of IVE [29,30]. However, the fact that our IVE estimates were constant in most seasons suggests that this particular source of error is negligible. Nevertheless, we cannot rule out that vaccination affected the severity of influenza disease and that consequently unvaccinated individuals were more likely to be hospitalised and tested than vaccinated individuals, despite the successful control for imbalances in healthcare utilisation.

## Conclusion

Although influenza vaccination does not provide perfect protection, it undoubtedly reduces the risk of severe influenza disease in vaccinated elderly people. However, vaccination does not prevent other acute respiratory diseases. When estimating IVE in the elderly population in Finland using a register-based cohort study design, individual-level data about age, sex, 1-year vaccination history and 5-year hospital care utilisation proved sufficient for the control of confounding.

## Supplementary Data

316000 Supplement
