# Supplementary material for: Spotlight influenza: Estimation of influenza vaccine effectiveness in elderly people with assessment of residual confounding by negative control outcomes, Finland, 2012/13 to 2019/20
Source: Euro Surveill. 2021 Sep 9;26(36):2100054. doi: 10.2807/1560-7917.ES.2021.26.36.2100054 (PMC8431990; doi:10.2807/1560-7917.ES.2021.26.36.2100054)

This supplementary material is hosted by Eurosurveillance as supporting information alongside the article **Spotlight influenza: Estimation of influenza vaccine effectiveness in elderly people with assessment of residual confounding by negative control outcomes, Finland, 2012/13 to 2019/20**, on behalf of the authors, who remain responsible for the accuracy and appropriateness of the content. The same standards for ethics, copyright, attributions and permissions as for the article apply. Supplements are not edited by Eurosurveillance and the journal is not responsible for the maintenance of any links or email addresses provided therein.

Supplementary Table S1: Enrollment of the study cohorts, 2012/13 to 2019/20 Individuals who had changed their municipality of residence in the five years prior to season onset or later as well as individuals who lived abroad or outside the National Vaccination Register's catchment area were excluded from enrollment. In addition, individuals who had participated in a randomized clinical trial evaluating the relative effectiveness of a high-dose quadrivalent influenza vaccine in 2019/20 were excluded in that season.

	Season 2012/13	Season 2013/14	Season 2014/15	Season 2015/16
**Inclusion criteria**				
Year of birth	1912–1947	1913–1948	1914–1949	1915–1950
Date of death	$\geq$ 01 Oct 2012	$\geq$ 01 Oct 2013	$\geq$ 01 Oct 2014	$\geq$ 01 Oct 2015
**Target population**				
Population count	1119579	1116115	1137265	1168742
**Exclusion criteria**				
Change of municipality	260193	229151	145652	140883
Residence outside Finland	12719	12571	13279	14483
Residence outside catchment area	43014	43521	26476	21444
**Study population**				
Population count	803653	830872	951858	991932

	Season 2016/17	Season 2017/18	Season 2018/19	Season 2019/20
**Inclusion criteria**				
Year of birth	1916–1951	1917–1952	1918–1953	1919–1954
Date of death	$\geq$ 01 Oct 2016	$\geq$ 01 Oct 2017	$\geq$ 01 Oct 2018	$\geq$ 01 Oct 2019
**Target population**				
Population count	1196064	1224882	1251024	1278503
**Exclusion criteria**				
Change of municipality	133832	129718	106287	99877
Residence outside Finland	15713	17080	18822	21134
Residence outside catchment area	21697	8187	2	19956
Participation in clinical trial	0	0	0	30310
**Study population**				
Population count	1024822	1069897	1125913	1107226

Supplementary Table S2: ICD-10 diagnostic codes Blocks, categories and subcategories of ICD-10 codes that were considered as indicators for the presence of underlying chronic conditions. Eighty-five percent of the identified chronically diseased study subjects had at least one of the conditions marked with a single asterisk. Ninety-five percent of the identified chronically diseased study subjects had at least one of the conditions marked with a double asterisk.

ICD-10	Underlying chronic condition		
A15–A16	Respiratory tuberculosis		
B20–B24	Human immunodeficiency virus disease		
C00–C97	Malignant neoplasms	*	**
D37–D48	Neoplasms of uncertain or unknown behaviour		**
D50–D53	Nutritional anaemias		
D55–D59	Haemolytic anaemias		
D60–D64	Aplastic and other anaemias		
D80–D84	Immunodeficiencies		
D89	Other disorders involving the immune mechanism, not elsewhere classified		
E10–E14	Diabetes mellitus	*	**
E66	Obesity		
E84.0	Cystic fibrosis with pulmonary manifestations		
F00–F03	Dementia	*	**
G30–G31	Other degenerative diseases of the nervous system		**
I00–I02	Acute rheumatic fever		
I05–I09	Chronic rheumatic heart diseases		
I11–I13	Hypertensive heart and/or renal disease		
I20–I25	Ischaemic heart diseases	*	**
I26–I28	Pulmonary heart disease and diseases of pulmonary circulation		
I30–I52	Other forms of heart disease	*	**
I60–I69	Cerebrovascular diseases	*	**
I70	Atherosclerosis		**
J40–J47	Chronic lower respiratory diseases	*	**
J60–J70	Lung diseases due to external agents		
J80–J84	Other respiratory diseases principally affecting the interstitium		
J96.1	Chronic respiratory failure		
J96.9	Respiratory failure, unspecified		
K70–K77	Diseases of liver		
M05–M08	Arthritis	*	**
M13	Other arthritis		
M30–M36	Systemic connective tissue disorders		**
M45	Ankylosing spondylitis		
N00–N08	Glomerular diseases		
N10–N16	Renal tubulo-interstitial diseases		**
N18–N19	Renal failure		**
N25–N27	Other disorders of kidney and ureter		
Q20–Q28	Congenital malformations of the circulatory system		
Q30–Q34	Congenital malformations of the respiratory system		
Z85	Personal history of malignant neoplasm		
Z94	Transplanted organ and tissue status		

Supplementary Table S3: Enrollment of the study cohorts, 2013 to 2019 Individuals who had changed their municipality of residence in the five years prior to the preceding season's onset or later as well as individuals who lived abroad or outside the National Vaccination Register's catchment area were excluded from enrollment. In addition, individuals who had been vaccinated against influenza or hospitalized with an acute respiratory infection in the month prior to off-season onset were excluded.

	Off-season 2013	Off-season 2014	Off-season 2015	Off-season 2016
**Inclusion criteria**				
Year of birth	1912–1947	1913–1948	1914–1949	1915–1950
Date of death	$\geq$ 01 Jul 2013	$\geq$ 01 Jul 2014	$\geq$ 01 Jul 2015	$\geq$ 01 Jul 2016
**Target population**				
Population count	1053666	1068318	1102206	1133056
**Exclusion criteria**				
Change of municipality	227186	211334	142172	137300
Residence outside Finland	11991	12300	13254	14464
Residence outside catchment area	41334	42012	25514	20728
Recent influenza vaccination	20	22	65	2
Recent hospitalization	2585	2724	3223	3310
**Study population**				
Population count	770550	799926	917978	957252

	Off-season 2017	Off-season 2018	Off-season 2019
**Inclusion criteria**			
Year of birth	1916–1951	1917–1952	1918–1953
Date of death	$\geq$ 01 Jul 2017	$\geq$ 01 Jul 2018	$\geq$ 01 Jul 2019
**Target population**			
Population count	1159274	1188242	1214824
**Exclusion criteria**			
Change of municipality	130112	125902	103307
Residence outside Finland	15695	17054	18796
Residence outside catchment area	21009	7905	2
Recent influenza vaccination	12	39	112
Recent hospitalization	3406	3385	2764
**Study population**			
Population count	989040	1033957	1089843

Supplementary Table S4: Distribution of covariates, 2012/13 to 2019/20 Distribution of covariates by the study subjects' vaccination status at the end of the study period.

	Season 2012/13				Season 2013/14				Season 2014/15				Season 2015/16			
	Unvaccinated		Vaccinated		Unvaccinated		Vaccinated		Unvaccinated		Vaccinated		Unvaccinated		Vaccinated	
	n	%	n	%	n	%	n	%	n	%	n	%	n	%	n	%
**Age in years**																
65–69	178741	35	69871	24	177975	37	89612	26	210830	37	103257	27	200424	35	122255	29
70–74	112105	22	70109	24	103554	21	80884	23	118349	21	88346	23	122117	22	106789	25
75–79	85136	17	62101	21	80629	17	72164	21	93997	17	81703	21	89999	16	86774	20
80–84	68592	14	50564	17	62257	13	54930	16	70657	12	59106	15	70342	12	61588	14
85–100	63184	12	43250	15	61314	13	47553	14	74942	13	50671	13	81846	14	49798	12
**Sex**																
Female	296345	58	170025	57	281666	58	197968	57	326949	57	217473	57	324433	57	239588	56
Male	211413	42	125870	43	204063	42	147175	43	241826	43	165610	43	240295	43	187616	44
**Previously vaccinated**																
No	420272	83	47818	16	444660	92	90585	26	494775	87	70876	19	495760	88	106978	25
Yes	87486	17	248077	84	41069	8	254558	74	74000	13	312207	81	68968	12	320226	75
**Chronically diseased**																
No	389675	77	207558	70	375468	77	245074	71	437901	77	271445	71	430916	76	306702	72
Yes	118083	23	88337	30	110261	23	100069	29	130874	23	111638	29	133812	24	120502	28
**Nights hospitalized**																
0	264058	52	131763	45	257436	53	161333	47	302613	53	183618	48	298106	53	214658	50
1–5	89177	18	58136	20	85660	18	69943	20	101579	18	80119	21	99764	18	92276	22
6–20	76244	15	53349	18	69810	14	59421	17	80041	14	64601	17	77717	14	69638	16
≥ 21	78279	15	52647	18	72823	15	54446	16	84542	15	54745	14	89141	16	50632	12

	Season 2016/17				Season 2017/18				Season 2018/19				Season 2019/20			
	Unvaccinated		Vaccinated		Unvaccinated		Vaccinated		Unvaccinated		Vaccinated		Unvaccinated		Vaccinated	
	n	%	n	%	n	%	n	%	n	%	n	%	n	%	n	%
**Age in years**																
65–69	185486	34	135012	28	189339	34	132891	26	186516	33	139149	25	184143	32	123460	23
70–74	120621	22	122446	25	138741	25	138552	27	147745	26	159116	29	157493	27	152807	29
75–79	89794	17	101493	21	89158	16	101175	20	88375	16	109558	20	90185	16	102114	19
80–84	65306	12	68455	14	66374	12	72604	14	68623	12	80605	14	70810	12	79750	15
85–100	78645	15	57564	12	78156	14	62907	12	78693	14	67533	12	77255	13	69209	13
**Sex**																
Female	307812	57	272441	56	316981	56	286410	56	318908	56	313148	56	323033	56	299033	57
Male	232040	43	212529	44	244787	44	221719	44	251044	44	242813	44	256853	44	228307	43
**Previously vaccinated**																
No	474541	88	114116	24	479801	85	90770	18	493504	87	105016	19	488400	84	88697	17
Yes	65311	12	370854	76	81967	15	417359	82	76448	13	450945	81	91486	16	438643	83
**Chronically diseased**																
No	409810	76	345508	71	427390	76	362218	71	433930	76	398372	72	466449	80	398186	76
Yes	130042	24	139462	29	134378	24	145911	29	136022	24	157589	28	113437	20	129154	24
**Nights hospitalized**																
0	287512	53	246641	51	304289	54	258451	51	310130	54	286292	51	331447	57	278011	53
1–5	96114	18	106070	22	101935	18	111863	22	104478	18	123339	22	104938	18	113832	22
6–20	73370	14	76290	16	75024	13	79361	16	75606	13	84780	15	72762	13	77346	15
≥ 21	82856	15	55969	12	80520	14	58454	12	79738	14	61550	11	70739	12	58151	11

Supplementary Table S5: Distribution of administered vaccine brands, 2012/13 to 2019/20
Vaccine brands that were not available in the Finnish vaccination program are summarized as “Others”.

Vaccine brand	n	%
**Season 2012/13**		
Fluarix (GlaxoSmithKline)	234697	79
Vaxigrip (Sanofi Pasteur)	40864	14
Others	2337	1
Unknown	17997	6
**Season 2013/14**		
Fluarix (GlaxoSmithKline)	308874	89
Vaxigrip (Sanofi Pasteur)	33329	10
Others	360	0
Unknown	2580	1
**Season 2014/15**		
Fluarix (GlaxoSmithKline)	355379	93
Vaxigrip (Sanofi Pasteur)	27166	7
Others	170	0
Unknown	368	0
**Season 2015/16**		
Fluarix (GlaxoSmithKline)	387080	91
Vaxigrip (Sanofi Pasteur)	39888	9
Others	154	0
Unknown	82	0
**Season 2016/17**		
Influvac (Abbott)	484459	100
Others	457	0
Unknown	54	0
**Season 2017/18**		
Agrippal (Seqirus)	11511	2
Influvac (Abbott)	495358	97
Others	1220	0
Unknown	40	0
**Season 2018/19**		
Vaxigrip Tetra (Sanofi Pasteur)	555618	100
Others	211	0
Unknown	132	0
**Season 2019/20**		
Vaxigrip Tetra (Sanofi Pasteur)	516730	98
Others	5255	1
Unknown	5355	1

Supplementary Table S6: Number of hospitalized laboratory-confirmed cases, 2012/13 to 2018/19 Number and percentage of laboratory-confirmed influenza cases hospitalized within seven days since the influenza-positive specimen was sampled.

	Unvaccinated				Fully vaccinated				Top 5 ICD-10 diagnostic codes in cases without J09–J10				
	Hospitalized		Hospitalized with J09–J10		Hospitalized		Hospitalized with J09–J10						
Season	n	%	n	%	n	%	n	%	1st	2nd	3rd	4th	5th
2012/13	370	84	216	49	165	89	104	56	J18.9	I10	I48	I50.9	J06.9
2013/14	400	85	267	57	184	90	129	63	J18.9	I10	I50.9	I48	J06.9
2014/15	1553	88	1017	57	885	86	621	60	J18.9	I10	I48	I50.9	J06.9
2015/16	1209	89	889	66	443	89	337	68	J18.9	I10	I48	J06.9	I50.9
2016/17	4003	86	3103	66	2615	87	2051	68	J18.9	I10	I48	J06.9	I50.9
2017/18	5600	84	4360	66	4498	85	3552	67	J18.9	I10	I48	J06.9	I50.9
2018/19	1653	61	1269	47	1243	60	983	47	J18.9	I48	I10	I50.9	J06.9

I10 : Essential (primary) hypertension

I48 : Atrial fibrillation and flutter

I50.9 : Heart failure, unspecified

J06.9 : Acute upper respiratory infection, unspecified

J09 : Influenza due to identified zoonotic or pandemic influenza virus

J10 : Influenza due to identified seasonal influenza virus

J18.9 : Pneumonia, unspecified

Supplementary Table S7: Influenza hazard ratio, 2012/13 to 2019/20 Point and 95% confidence interval estimates for crude and adjusted hazard ratios comparing the hazard of laboratory-confirmed influenza in fullly vaccinated study subjects with the corresponding hazard in the unvaccinated.

Season	Crude hazard ratio	Adjusted hazard ratio
2012/13	0.72 (0.61–0.86)	0.69 (0.56–0.85)
2013/14	0.65 (0.55–0.76)	0.55 (0.44–0.68)
2014/15	0.88 (0.81–0.95)	0.78 (0.71–0.86)
2015/16	0.50 (0.45–0.56)	0.52 (0.46–0.59)
2016/17	0.78 (0.75–0.82)	0.76 (0.72–0.81)
2017/18	0.88 (0.84–0.91)	0.84 (0.81–0.88)
2018/19	0.80 (0.75–0.85)	0.74 (0.69–0.80)
2019/20	0.82 (0.74–0.90)	0.76 (0.67–0.85)

Supplementary Table S8: Vaccine effectiveness by previous vaccination status, 2012/13 to 2019/20 Influenza vaccine effectiveness against laboratory-confirmed influenza by vaccination status at the end of the previous season.

		Unvaccinated		Fully vaccinated		Vaccine effectiveness, %	
Previously vaccinated	N	Cases	Attack rate	Cases	Attack rate	Estimate	95% CI
**Season 2012/13**							
No	468090	372	90	28	61	48	24 to 65
Yes	335563	69	81	157	65	19	-8 to 39
**Season 2013/14**							
No	535245	422	96	39	48	56	39 to 68
Yes	295627	48	116	166	68	33	8 to 52
**Season 2014/15**							
No	565651	1435	294	197	295	25	13 to 36
Yes	386207	335	462	830	272	19	8 to 29
**Season 2015/16**							
No	602738	1185	241	101	141	61	52 to 68
Yes	389194	172	247	394	127	33	20 to 44
**Season 2016/17**							
No	588657	3779	800	667	670	26	20 to 32
Yes	436165	891	1241	2339	674	23	17 to 29
**Season 2017/18**							
No	570571	5367	1139	1064	1210	14	8 to 19
Yes	499326	1283	1601	4199	1020	17	12 to 23
**Season 2018/19**							
No	598520	2167	447	403	394	23	14 to 31
Yes	527393	523	700	1685	380	28	20 to 35
**Season 2019/20**							
No	577097	842	172	139	185	16	-1 to 30
Yes	530129	253	231	540	143	30	18 to 40

The attack rate is presented as the cumulative risk at the end of the study period multiplied by 10^5.

Supplementary Table S9: Vaccine effectiveness by virus type, 2012/13 to 2019/20 Influenza vaccine effectiveness against laboratory-confirmed influenza A and laboratory-confirmed influenza B.

		Unvaccinated		Fully vaccinated		Vaccine effectiveness, %	
Virus type	N	Cases	Attack rate	Cases	Attack rate	Estimate	95% CI
**Season 2012/13**							
Influenza A	803653	370	75	166	57	28	9 to 42
Influenza B	803653	73	15	20	7	50	9 to 73
**Season 2013/14**							
Influenza A	830872	442	92	196	60	45	31 to 56
Influenza B	830872	28	6	10	3	52	-22 to 81
**Season 2014/15**							
Influenza A	951858	1281	228	819	221	16	6 to 25
Influenza B	951858	502	90	212	56	38	25 to 50
**Season 2015/16**							
Influenza A	991932	1128	200	388	103	49	41 to 56
Influenza B	991932	239	43	112	27	42	24 to 56
**Season 2016/17**							
Influenza A	1024822	4581	840	2943	649	24	20 to 28
Influenza B	1024822	100	19	66	23	7	-37 to 37
**Season 2017/18**							
Influenza A	1069897	3404	617	3129	626	9	3 to 14
Influenza B	1069897	3308	600	2177	436	23	17 to 28
**Season 2018/19**							
Influenza A	1125913	2681	479	2078	381	26	20 to 31
Influenza B	1125913	13	2	13	2	17	-118 to 69
**Season 2019/20**							
Influenza A	1107226	1046	173	654	144	24	14 to 33
Influenza B	1107226	50	9	25	5	28	-31 to 60

The attack rate is presented as the cumulative risk at the end of the study period multiplied by 10^5.

Supplementary Table S10: Acute respiratory infection hazard ratio, 2012/13 to 2018/19 Point and 95% confidence interval estimates for crude and adjusted hazard ratios comparing the hazard of hospitalization for acute respiratory infection in fully vaccinated study subjects with the corresponding hazard in the unvaccinated.

Season	Crude hazard ratio	Adjusted hazard ratio
2012/13	1.32 (1.28–1.36)	1.01 (0.98–1.05)
2013/14	1.24 (1.20–1.28)	0.94 (0.90–0.98)
2014/15	1.17 (1.14–1.20)	0.96 (0.93–0.99)
2015/16	1.04 (1.02–1.07)	0.98 (0.95–1.01)
2016/17	1.07 (1.04–1.09)	1.00 (0.97–1.03)
2017/18	1.06 (1.04–1.09)	0.97 (0.94–1.00)
2018/19	1.10 (1.07–1.13)	1.02 (0.99–1.06)

Supplementary Table S11: Distribution of covariates, 2013 to 2019 Distribution of covariates by the study subjects' vaccination status at off-season onset.

	Off-season 2013				Off-season 2014				Off-season 2015				Off-season 2016	
	Unvaccinated		Vaccinated		Unvaccinated		Vaccinated		Unvaccinated		Vaccinated		Unvaccinated	
	n	%	n	%	n	%	n	%	n	%	n	%	n	%
**Age in years**														
65–69	176015	36	69068	24	175371	38	88759	26	208050	38	102289	27	197609	37
70–74	109380	23	69068	24	101076	22	79821	24	115825	21	87219	23	119395	22
75–79	81659	17	60574	21	77499	17	70674	21	90609	17	80085	21	86424	16
80–84	63897	13	48363	17	58107	13	52810	16	66094	12	57153	15	65643	12
85–100	53600	11	38926	14	52488	11	43321	13	64189	12	46465	12	69902	13
**Sex**														
Female	283743	59	164722	58	270228	58	192834	57	313613	58	212411	57	310378	58
Male	200808	41	121277	42	194313	42	142551	43	231154	42	160800	43	228595	42
**Previously vaccinated**														
No	404429	83	45706	16	428894	92	88047	26	478583	88	68293	18	477066	89
Yes	80122	17	240293	84	35647	8	247338	74	66184	12	304918	82	61907	11
**Chronically diseased**														
No	380043	78	203312	71	366607	79	240920	72	427549	78	267370	72	419628	78
Yes	104508	22	82687	29	97934	21	94465	28	117218	22	105841	28	119345	22
**Nights hospitalized**														
0	261211	54	130566	46	254783	55	160028	48	299469	55	182295	49	294699	55
1–5	87529	18	57260	20	84018	18	69028	21	99650	18	79178	21	97598	18
6–20	72451	15	51481	18	66315	14	57568	17	76039	14	62683	17	73413	14
$\geq$ 21	63360	13	46692	16	59425	13	48761	15	69609	13	49055	13	73263	14

	Off-season 2016		Off-season 2017				Off-season 2018				Off-season 2019			
	Vaccinated		Unvaccinated		Vaccinated		Unvaccinated		Vaccinated		Unvaccinated		Vaccinated	
	n	%	n	%	n	%	n	%	n	%	n	%	n	%
**Age in years**														
65–69	121267	29	182915	36	133947	28	186877	35	131826	27	184148	34	138099	25
70–74	105544	25	117822	23	121063	26	135796	25	136998	28	144559	27	157511	29
75–79	85285	20	86271	17	99668	21	85712	16	99427	20	85037	16	107781	20
80–84	59836	14	60823	12	66335	14	61980	12	70358	14	64316	12	78368	14
85–100	46347	11	66945	13	53251	11	66905	12	58078	12	67402	12	62622	12
**Sex**														
Female	235254	56	294111	57	267155	56	303703	57	280667	57	305688	56	307381	56
Male	183025	44	220665	43	207109	44	233567	43	216020	43	239774	44	237000	44
**Previously vaccinated**														
No	104449	25	455239	88	110625	23	461713	86	87495	18	475870	87	101900	19
Yes	313830	75	59537	12	363639	77	75557	14	409192	82	69592	13	442481	81
**Chronically diseased**														
No	303107	72	398682	77	341095	72	416582	78	357437	72	422964	78	393553	72
Yes	115172	28	116094	23	133169	28	120688	22	139250	28	122498	22	150828	28
**Nights hospitalized**														
0	213313	51	284201	55	245058	52	300898	56	256766	52	306617	56	284625	52
1–5	91225	22	93941	18	104840	22	99788	19	110480	22	102273	19	121896	22
6–20	67688	16	69060	13	74057	16	70621	13	76977	15	71182	13	82288	15
$\geq$ 21	46053	11	67574	13	50309	11	65963	12	52464	11	65390	12	55572	10

Supplementary Table S12: Logarithm of the acute respiratory infection hazard ratio, 2013 to 2019 Natural logarithm of the covariate-adjusted hazard ratio comparing the hazard of hospitalization for acute respiratory infection in the vaccinated with the corresponding hazard in the unvaccinated. The entry in the first column in each row indicates which covariates and interaction terms were included in the regression model. Interaction terms are marked with the colon (:) symbol.

AY5: Age in years (65–69; 70–74; 75–79; 80–84; 85–100)
AY9: Age in years (65; 66; 67; 68–69; 70–72; 73–75; 76–79; 80–84; 85–100)
PV1: Vaccinated at the end of the previous season (No; Yes)
PV3: Number of influenza vaccinations received in the previous three seasons (0; 1–2; 3)
CD1: Presence of underlying chronic conditions based on one-year history of inpatient and outpatient hospital visits prior to season onset (No; Yes)
CD3: Presence of underlying chronic conditions based on three-year history of inpatient and outpatient hospital visits prior to season onset (No; Yes)
CD5: Presence of underlying chronic conditions based on five-year history of inpatient and outpatient hospital visits prior to season onset (No; Yes)
NH1: Number of nights hospitalized in the year prior to season onset (0; 1–5; 6–20; $\geq$ 21)
NH3: Number of nights hospitalized in the three years prior to season onset (0; 1–5; 6–20; $\geq$ 21)
NH5: Number of nights hospitalized in the five years prior to season onset (0; 1–5; 6–20; $\geq$ 21)

	Off-season							
	2013	2014	2015	2016	2017	2018	2019	Rank
AY9 + Sex + PV1 + CD1 + NH5 + AY9:PV1 + CD1:PV1	0.073	0.001	0.011	-0.005	-0.006	0.000	0.069	1
AY9 + Sex + PV1 + CD1 + NH5 + AY9:PV1	0.072	0.001	0.008	-0.009	-0.007	-0.001	0.065	2
AY5 + Sex + PV1 + CD1 + NH5 + AY5:PV1 + CD1:PV1	0.073	0.001	0.011	-0.005	-0.007	0.000	0.069	3
AY5 + Sex + PV1 + CD1 + NH5 + AY5:PV1	0.072	0.001	0.008	-0.010	-0.007	-0.001	0.065	4
AY9 + Sex + PV1 + CD3 + NH5 + AY9:PV1	0.073	0.000	0.016	-0.004	-0.007	-0.001	0.067	5
AY5 + Sex + PV1 + CD3 + NH5 + AY5:PV1	0.073	0.000	0.016	-0.004	-0.007	-0.001	0.067	6
AY5 + Sex + PV1 + CD3 + NH5 + AY5:PV1 + CD3:PV1	0.072	0.000	0.019	0.000	-0.005	0.001	0.071	7
AY9 + Sex + PV1 + CD3 + NH5 + AY9:PV1 + CD3:PV1	0.073	0.000	0.019	0.001	-0.005	0.001	0.071	8
AY9 + Sex + PV1 + CD1 + NH3 + AY9:PV1	0.079	0.001	0.004	-0.018	-0.006	0.000	0.061	9
AY5 + Sex + PV1 + CD1 + NH3 + AY5:PV1	0.079	0.001	0.004	-0.018	-0.006	0.000	0.061	10
AY9 + Sex + PV1 + CD5 + NH5 + AY9:PV1	0.074	0.002	0.018	-0.002	-0.007	0.000	0.068	11
AY9 + Sex + PV1 + CD1 + NH3 + AY9:PV1 + CD1:PV1	0.080	0.000	0.007	-0.012	-0.005	0.003	0.066	12
AY5 + Sex + PV1 + CD1 + NH3 + AY5:PV1 + CD1:PV1	0.080	0.000	0.007	-0.012	-0.005	0.003	0.066	13
AY5 + Sex + PV1 + CD5 + NH5 + AY5:PV1	0.074	0.002	0.018	-0.002	-0.007	0.000	0.068	14
AY9 + Sex + PV1 + CD1 + NH5	0.076	0.004	0.013	-0.003	-0.003	0.006	0.069	15
AY5 + Sex + PV1 + CD5 + NH3 + AY5:PV1 + CD5:PV1	0.076	-0.002	0.013	-0.011	-0.008	0.000	0.064	16
AY9 + Sex + PV1 + CD5 + NH3 + AY9:PV1 + CD5:PV1	0.076	-0.002	0.013	-0.011	-0.007	0.000	0.065	17
AY9 + Sex + PV1 + CD5 + NH3 + AY9:PV1	0.076	-0.003	0.009	-0.013	-0.010	-0.002	0.060	18
AY5 + Sex + PV1 + CD1 + NH5	0.076	0.004	0.013	-0.003	-0.003	0.006	0.069	19
AY9 + Sex + PV1 + CD3 + NH3 + AY9:PV1	0.079	-0.002	0.011	-0.013	-0.008	0.000	0.062	20
AY5 + Sex + PV1 + CD3 + NH3 + AY5:PV1 + CD3:PV1	0.078	-0.002	0.014	-0.008	-0.005	0.002	0.066	21
AY5 + Sex + PV1 + CD5 + NH3 + AY5:PV1	0.076	-0.003	0.009	-0.014	-0.010	-0.002	0.060	22
AY9 + Sex + PV1 + CD3 + NH3 + AY9:PV1 + CD3:PV1	0.079	-0.002	0.014	-0.008	-0.005	0.002	0.067	23
AY5 + Sex + PV1 + CD3 + NH3 + AY5:PV1	0.079	-0.002	0.010	-0.014	-0.008	0.000	0.062	24
AY9 + Sex + PV1 + CD1 + NH5 + CD1:PV1	0.076	0.003	0.015	0.002	-0.003	0.006	0.072	25
AY5 + Sex + PV1 + CD1 + NH5 + CD1:PV1	0.076	0.003	0.015	0.002	-0.003	0.006	0.072	26
AY9 + Sex + PV1 + CD5 + NH3	0.080	-0.001	0.013	-0.007	-0.007	0.005	0.064	27
AY5 + Sex + PV1 + CD5 + NH3	0.080	-0.001	0.014	-0.007	-0.007	0.005	0.065	28
AY5 + Sex + PV1 + CD5 + NH5 + AY5:PV1 + CD5:PV1	0.074	0.004	0.022	0.000	-0.005	0.002	0.072	29
AY9 + Sex + PV1 + CD5 + NH5 + AY9:PV1 + CD5:PV1	0.074	0.004	0.022	0.001	-0.005	0.002	0.072	30
AY9 + Sex + PV1 + CD5 + NH3 + CD5:PV1	0.079	0.000	0.017	-0.005	-0.004	0.006	0.068	31
AY9 + Sex + PV1 + CD3 + NH3 + CD3:PV1	0.081	0.000	0.018	-0.002	-0.001	0.008	0.070	32
AY5 + Sex + PV1 + CD5 + NH3 + CD5:PV1	0.079	0.001	0.017	-0.005	-0.005	0.006	0.068	33
AY5 + Sex + PV1 + CD3 + NH3 + CD3:PV1	0.081	0.001	0.018	-0.002	-0.001	0.008	0.070	34
AY9 + Sex + PV1 + CD1 + NH3 + CD1:PV1	0.083	0.002	0.011	-0.005	-0.001	0.010	0.070	35
AY9 + Sex + PV1 + CD3 + NH3	0.083	0.001	0.015	-0.006	-0.004	0.008	0.066	36
AY9 + Sex + PV1 + CD1 + NH3	0.083	0.004	0.009	-0.010	-0.001	0.009	0.066	37
AY9 + Sex + PV1 + CD3 + NH5	0.077	0.002	0.020	0.002	-0.004	0.006	0.070	38
AY5 + Sex + PV1 + CD1 + NH3 + CD1:PV1	0.083	0.003	0.011	-0.005	-0.001	0.010	0.070	39
AY5 + Sex + PV1 + CD3 + NH3	0.083	0.001	0.015	-0.006	-0.004	0.008	0.066	40

Supplementary Table 12 continued

	Off-season							
	2013	2014	2015	2016	2017	2018	2019	Rank
AY5 + Sex + PV1 + CD3 + NH5	0.077	0.002	0.021	0.002	-0.004	0.006	0.071	41
AY5 + Sex + PV1 + CD1 + NH3	0.083	0.004	0.009	-0.010	-0.001	0.009	0.066	42
AY9 + Sex + PV1 + CD3 + NH5 + CD3:PV1	0.075	0.002	0.023	0.007	-0.002	0.006	0.074	43
AY5 + Sex + PV1 + CD3 + NH5 + CD3:PV1	0.075	0.002	0.023	0.007	-0.002	0.006	0.074	44
AY9 + Sex + PV1 + CD5 + NH5	0.078	0.005	0.023	0.004	-0.004	0.006	0.071	45
AY5 + Sex + PV1 + CD5 + NH5	0.078	0.005	0.023	0.004	-0.004	0.007	0.072	46
AY9 + Sex + PV1 + CD5 + NH5 + CD5:PV1	0.077	0.006	0.026	0.006	-0.002	0.007	0.075	47
AY5 + Sex + PV1 + CD5 + NH5 + CD5:PV1	0.077	0.006	0.026	0.006	-0.002	0.007	0.075	48
AY5 + PV1 + CD5 + NH3 + AY5:PV1	0.092	0.006	0.022	0.004	0.000	0.006	0.070	49
AY9 + PV1 + CD5 + NH3 + AY9:PV1	0.092	0.006	0.022	0.005	0.001	0.007	0.070	50
AY5 + PV1 + CD1 + NH3 + AY5:PV1	0.095	0.010	0.016	-0.001	0.004	0.009	0.070	51
AY9 + PV1 + CD1 + NH3 + AY9:PV1	0.095	0.010	0.016	0.000	0.004	0.009	0.070	52
Sex + PV1 + CD1 + NH5	0.089	0.000	0.003	-0.036	-0.021	0.005	0.054	53
Sex + PV1 + CD1 + NH5 + CD1:PV1	0.087	-0.003	0.003	-0.033	-0.023	0.003	0.055	54
Sex + PV1 + CD3 + NH5 + CD3:PV1	0.085	-0.004	0.011	-0.028	-0.021	0.002	0.057	55
AY5 + PV1 + CD3 + NH3 + AY5:PV1	0.095	0.007	0.023	0.004	0.002	0.008	0.071	56
AY5 + PV1 + CD1 + NH5 + AY5:PV1	0.088	0.011	0.020	0.008	0.003	0.007	0.074	57
Sex + PV1 + CD5 + NH5	0.088	-0.001	0.012	-0.028	-0.022	0.004	0.057	58
Sex + PV1 + CD3 + NH5	0.088	-0.003	0.009	-0.030	-0.022	0.004	0.056	59
AY9 + PV1 + CD3 + NH3 + AY9:PV1	0.095	0.007	0.023	0.004	0.003	0.008	0.071	60
Sex + PV1 + CD5 + NH5 + CD5:PV1	0.087	-0.001	0.013	-0.028	-0.021	0.003	0.058	61
AY9 + PV1 + CD5 + NH1 + AY9:PV1	0.100	0.000	-0.001	-0.028	-0.012	0.008	0.063	62
AY9 + PV1 + CD1 + NH5 + AY9:PV1	0.088	0.011	0.020	0.008	0.004	0.007	0.074	63
AY5 + PV1 + CD5 + NH1 + AY5:PV1	0.099	0.000	-0.001	-0.029	-0.013	0.008	0.063	64
PV1 + CD3 + NH5 + CD3:PV1	0.097	0.002	0.022	-0.009	-0.011	0.008	0.065	65
AY9 + PV1 + CD3 + NH1 + AY9:PV1 + CD3:PV1	0.101	0.000	0.002	-0.025	-0.009	0.011	0.067	66
AY5 + PV1 + CD3 + NH1 + AY5:PV1 + CD3:PV1	0.100	0.000	0.002	-0.025	-0.010	0.010	0.067	67
PV1 + CD1 + NH5 + CD1:PV1	0.099	0.004	0.013	-0.016	-0.013	0.008	0.063	68
AY9 + PV1 + CD3 + NH1 + AY9:PV1	0.101	-0.001	-0.001	-0.030	-0.012	0.009	0.063	69
PV1 + CD5 + NH3	0.102	-0.001	0.011	-0.024	-0.017	0.007	0.054	70
AY5 + PV1 + CD3 + NH1 + AY5:PV1	0.101	-0.001	-0.002	-0.030	-0.012	0.009	0.063	71
PV1 + CD3 + NH5	0.099	0.003	0.020	-0.012	-0.013	0.008	0.063	72
Sex + PV1 + CD5 + NH3	0.091	-0.008	0.001	-0.042	-0.027	0.002	0.047	73
Sex + PV1 + CD5 + NH3 + CD5:PV1	0.090	-0.007	0.003	-0.042	-0.026	0.001	0.049	74
PV1 + CD1 + NH5	0.099	0.006	0.012	-0.019	-0.012	0.009	0.060	75
PV1 + CD5 + NH3 + CD5:PV1	0.101	-0.001	0.014	-0.023	-0.016	0.007	0.057	76
AY9 + PV1 + CD5 + NH1 + AY9:PV1 + CD5:PV1	0.100	0.003	0.003	-0.026	-0.010	0.010	0.067	77
AY5 + PV1 + CD5 + NH1 + AY5:PV1 + CD5:PV1	0.100	0.002	0.003	-0.026	-0.011	0.010	0.067	78
AY9 + PV1 + CD5 + NH1	0.102	0.002	0.002	-0.023	-0.009	0.016	0.066	79
AY9 + PV1 + CD3 + NH1 + CD3:PV1	0.102	0.001	0.005	-0.019	-0.006	0.016	0.069	80
AY5 + PV1 + CD3 + NH1 + CD3:PV1	0.102	0.001	0.005	-0.019	-0.007	0.016	0.069	81
AY5 + PV1 + CD5 + NH3 + AY5:PV1 + CD5:PV1	0.092	0.008	0.026	0.007	0.003	0.009	0.075	82
AY5 + PV1 + CD5 + NH1	0.102	0.002	0.002	-0.023	-0.009	0.016	0.066	83
AY9 + Sex + PV1 + CD5 + NH1 + CD5:PV1	0.087	-0.005	-0.005	-0.038	-0.016	0.008	0.060	84
AY5 + Sex + PV1 + CD5 + NH1 + CD5:PV1	0.087	-0.005	-0.006	-0.038	-0.016	0.008	0.060	85
AY9 + PV1 + CD3 + NH1	0.104	0.001	0.002	-0.023	-0.008	0.017	0.066	86
Sex + PV1 + CD3 + NH3 + CD3:PV1	0.093	-0.007	0.004	-0.039	-0.023	0.003	0.051	87
PV1 + CD3 + NH3 + CD3:PV1	0.104	-0.001	0.014	-0.021	-0.014	0.009	0.058	88
AY9 + Sex + PV1 + CD3 + NH1 + CD3:PV1	0.087	-0.008	-0.006	-0.036	-0.015	0.008	0.060	89
AY5 + Sex + PV1 + CD3 + NH1 + CD3:PV1	0.087	-0.008	-0.006	-0.036	-0.015	0.008	0.061	90
AY9 + PV1 + CD5 + NH3 + AY9:PV1 + CD5:PV1	0.092	0.008	0.026	0.008	0.003	0.009	0.075	91
AY5 + PV1 + CD3 + NH1	0.103	0.002	0.002	-0.024	-0.008	0.017	0.066	92
PV1 + CD5 + NH5	0.099	0.005	0.023	-0.009	-0.013	0.009	0.064	93
AY5 + PV1 + CD1 + NH3 + AY5:PV1 + CD1:PV1	0.096	0.010	0.019	0.005	0.005	0.012	0.075	94
AY9 + Sex + PV1 + CD5 + NH1 + AY9:PV1 + CD5:PV1	0.084	-0.007	-0.009	-0.044	-0.020	0.001	0.056	95
Sex + PV1 + CD1 + NH3	0.098	0.000	-0.001	-0.046	-0.021	0.007	0.050	96
AY5 + Sex + PV1 + CD5 + NH1 + AY5:PV1 + CD5:PV1	0.084	-0.007	-0.010	-0.045	-0.021	0.001	0.056	97
Sex + PV1 + CD3 + NH3	0.096	-0.005	0.004	-0.041	-0.024	0.005	0.049	98
AY9 + PV1 + CD1 + NH3 + AY9:PV1 + CD1:PV1	0.096	0.010	0.019	0.006	0.006	0.012	0.076	99
AY9 + PV1 + CD5 + NH3	0.095	0.008	0.025	0.010	0.002	0.012	0.072	100
AY5 + PV1 + CD5 + NH3	0.095	0.008	0.025	0.010	0.002	0.012	0.073	101
PV1 + CD3 + NH3	0.106	0.001	0.013	-0.024	-0.015	0.009	0.056	102
PV1 + CD5 + NH5 + CD5:PV1	0.098	0.006	0.025	-0.009	-0.011	0.009	0.067	103

Supplementary Table 12 continued

	Off-season							
	2013	2014	2015	2016	2017	2018	2019	Rank
AY9 + PV1 + CD5 + NH1 + CD5:PV1	0.102	0.004	0.006	-0.021	-0.007	0.016	0.069	104
AY5 + Sex + PV1 + CD5 + NH1	0.087	-0.007	-0.008	-0.039	-0.017	0.008	0.058	105
AY9 + Sex + PV1 + CD5 + NH1	0.087	-0.007	-0.009	-0.039	-0.017	0.008	0.058	106
AY9 + Sex + PV1 + CD3 + NH1 + AY9:PV1 + CD3:PV1	0.085	-0.010	-0.010	-0.043	-0.019	0.001	0.057	107
AY5 + PV1 + CD5 + NH1 + CD5:PV1	0.102	0.004	0.006	-0.021	-0.008	0.016	0.069	108
Sex + PV1 + CD1 + NH3 + CD1:PV1	0.097	-0.004	-0.001	-0.043	-0.022	0.007	0.052	109
AY5 + PV1 + CD1 + NH5 + AY5:PV1 + CD1:PV1	0.089	0.010	0.023	0.013	0.004	0.008	0.079	110
AY5 + Sex + PV1 + CD3 + NH1 + AY5:PV1 + CD3:PV1	0.084	-0.010	-0.011	-0.043	-0.020	0.001	0.057	111
AY5 + PV1 + CD3 + NH5 + AY5:PV1	0.089	0.008	0.029	0.013	0.003	0.007	0.077	112
AY9 + PV1 + CD1 + NH5 + AY9:PV1 + CD1:PV1	0.089	0.010	0.023	0.013	0.004	0.009	0.079	113
AY9 + PV1 + CD3 + NH5 + AY9:PV1	0.089	0.009	0.029	0.014	0.003	0.007	0.077	114
AY9 + Sex + PV1 + CD5 + NH1 + AY9:PV1	0.084	-0.009	-0.013	-0.047	-0.022	0.000	0.053	115
PV1 + CD1 + NH3 + CD1:PV1	0.108	0.003	0.008	-0.026	-0.013	0.012	0.058	116
AY5 + Sex + PV1 + CD3 + NH1	0.089	-0.007	-0.009	-0.040	-0.016	0.010	0.059	117
AY9 + Sex + PV1 + CD3 + NH1	0.089	-0.008	-0.009	-0.040	-0.016	0.010	0.058	118
AY5 + Sex + PV1 + CD5 + NH1 + AY5:PV1	0.084	-0.009	-0.013	-0.047	-0.022	-0.001	0.053	119
AY5 + PV1 + CD3 + NH3 + AY5:PV1 + CD3:PV1	0.094	0.008	0.027	0.010	0.005	0.011	0.077	120
AY9 + Sex + PV1 + CD3 + NH1 + AY9:PV1	0.085	-0.010	-0.013	-0.048	-0.021	0.000	0.053	121
PV1 + CD1 + NH3	0.109	0.006	0.007	-0.029	-0.013	0.012	0.055	122
AY5 + Sex + PV1 + CD3 + NH1 + AY5:PV1	0.085	-0.010	-0.014	-0.048	-0.022	0.000	0.053	123
AY9 + PV1 + CD3 + NH3 + AY9:PV1 + CD3:PV1	0.095	0.008	0.027	0.010	0.006	0.011	0.077	124
AY9 + PV1 + CD1 + NH3	0.098	0.013	0.019	0.006	0.007	0.016	0.073	125
AY5 + PV1 + CD1 + NH3	0.098	0.013	0.019	0.006	0.007	0.016	0.073	126
AY9 + Sex + PV1 + CD1 + NH1 + CD1:PV1	0.098	0.002	-0.007	-0.035	-0.010	0.016	0.066	127
AY9 + Sex + PV1 + CD1 + NH1 + AY9:PV1 + CD1:PV1	0.095	-0.001	-0.011	-0.043	-0.015	0.008	0.061	128
AY5 + Sex + PV1 + CD1 + NH1 + CD1:PV1	0.097	0.002	-0.006	-0.036	-0.010	0.016	0.066	129
AY5 + Sex + PV1 + CD1 + NH1 + AY5:PV1 + CD1:PV1	0.095	-0.001	-0.011	-0.044	-0.016	0.008	0.061	130
AY9 + PV1 + CD3 + NH3	0.098	0.009	0.026	0.010	0.005	0.014	0.074	131
AY9 + PV1 + CD1 + NH5	0.091	0.013	0.024	0.013	0.006	0.013	0.077	132
AY9 + Sex + PV1 + CD1 + NH1 + AY9:PV1	0.095	0.001	-0.013	-0.049	-0.017	0.005	0.056	133
AY5 + PV1 + CD3 + NH3	0.098	0.009	0.026	0.010	0.005	0.014	0.074	134
AY5 + PV1 + CD1 + NH5	0.091	0.013	0.024	0.013	0.006	0.013	0.077	135
AY5 + PV1 + CD5 + NH5 + AY5:PV1	0.090	0.011	0.032	0.016	0.003	0.008	0.078	136
AY5 + Sex + PV1 + CD1 + NH1 + AY5:PV1	0.095	0.001	-0.013	-0.049	-0.018	0.005	0.056	137
AY9 + PV1 + CD5 + NH5 + AY9:PV1	0.090	0.011	0.032	0.016	0.003	0.008	0.078	138
AY9 + Sex + PV1 + CD1 + NH1	0.099	0.005	-0.007	-0.040	-0.011	0.016	0.063	139
AY5 + PV1 + CD5 + NH3 + CD5:PV1	0.094	0.009	0.029	0.012	0.005	0.014	0.077	140
AY9 + PV1 + CD5 + NH3 + CD5:PV1	0.095	0.009	0.029	0.012	0.005	0.014	0.077	141
AY9 + PV1 + CD1 + NH1 + AY9:PV1 + CD1:PV1	0.111	0.009	0.000	-0.026	-0.006	0.017	0.071	142
AY5 + Sex + PV1 + CD1 + NH1	0.099	0.005	-0.007	-0.040	-0.011	0.016	0.063	143
AY5 + PV1 + CD1 + NH1 + AY5:PV1 + CD1:PV1	0.111	0.009	0.000	-0.026	-0.007	0.017	0.071	144
AY5 + Sex + PV1 + NH3 + AY5:PV1	0.097	0.021	0.026	0.000	0.009	0.015	0.073	145
AY9 + Sex + PV1 + NH3 + AY9:PV1	0.097	0.021	0.026	0.000	0.009	0.015	0.073	146
AY9 + PV1 + CD1 + NH1 + AY9:PV1	0.111	0.011	-0.002	-0.032	-0.008	0.014	0.065	147
AY5 + PV1 + CD1 + NH1 + AY5:PV1	0.111	0.011	-0.002	-0.032	-0.009	0.013	0.065	148
AY5 + PV1 + CD3 + NH5 + AY5:PV1 + CD3:PV1	0.088	0.009	0.032	0.019	0.006	0.009	0.081	149
AY9 + PV1 + CD3 + NH5 + AY9:PV1 + CD3:PV1	0.088	0.009	0.032	0.019	0.006	0.009	0.081	150
AY9 + PV1 + CD1 + NH1 + CD1:PV1	0.113	0.011	0.004	-0.019	-0.002	0.024	0.074	151
AY5 + PV1 + CD1 + NH1 + CD1:PV1	0.113	0.011	0.004	-0.019	-0.002	0.024	0.074	152
AY9 + PV1 + CD1 + NH5 + CD1:PV1	0.091	0.012	0.026	0.018	0.007	0.014	0.080	153
AY5 + PV1 + CD1 + NH5 + CD1:PV1	0.091	0.012	0.026	0.018	0.006	0.014	0.080	154
AY5 + PV1 + CD1 + NH3 + CD1:PV1	0.099	0.012	0.022	0.011	0.009	0.018	0.078	155
AY9 + PV1 + CD1 + NH3 + CD1:PV1	0.099	0.012	0.022	0.011	0.009	0.018	0.078	156
AY9 + PV1 + CD3 + NH5	0.092	0.010	0.032	0.018	0.005	0.013	0.078	157
AY9 + PV1 + CD1 + NH1	0.114	0.014	0.002	-0.024	-0.003	0.023	0.069	158
AY5 + PV1 + CD3 + NH5	0.092	0.011	0.032	0.019	0.005	0.013	0.079	159
AY5 + PV1 + CD1 + NH1	0.114	0.014	0.002	-0.024	-0.003	0.023	0.070	160
AY5 + PV1 + CD3 + NH3 + CD3:PV1	0.096	0.009	0.030	0.015	0.008	0.015	0.079	161
AY9 + PV1 + CD3 + NH3 + CD3:PV1	0.097	0.009	0.030	0.015	0.008	0.016	0.078	162
AY5 + PV1 + CD5 + NH5 + AY5:PV1 + CD5:PV1	0.090	0.013	0.035	0.019	0.005	0.010	0.082	163
AY9 + PV1 + CD5 + NH5 + AY9:PV1 + CD5:PV1	0.090	0.013	0.035	0.019	0.006	0.010	0.083	164
AY5 + Sex + PV1 + NH5 + AY5:PV1	0.091	0.023	0.033	0.009	0.008	0.014	0.078	165
AY9 + Sex + PV1 + NH5 + AY9:PV1	0.091	0.023	0.033	0.010	0.008	0.014	0.078	166

Supplementary Table 12 continued

	Off-season							
	2013	2014	2015	2016	2017	2018	2019	Rank
AY9 + PV1 + CD5 + NH5	0.093	0.013	0.035	0.021	0.005	0.013	0.080	167
AY5 + PV1 + CD5 + NH5	0.093	0.013	0.035	0.021	0.005	0.013	0.080	168
AY5 + PV1 + CD3 + NH5 + CD3:PV1	0.090	0.011	0.036	0.023	0.008	0.013	0.082	169
AY9 + PV1 + CD3 + NH5 + CD3:PV1	0.090	0.011	0.035	0.023	0.008	0.013	0.082	170
AY5 + PV1 + CD5 + NH5 + CD5:PV1	0.092	0.015	0.039	0.023	0.007	0.014	0.084	171
Sex + PV1 + NH5	0.110	0.024	0.030	-0.014	-0.003	0.022	0.070	172
AY9 + PV1 + CD5 + NH5 + CD5:PV1	0.092	0.015	0.038	0.023	0.008	0.014	0.084	173
Sex + PV1 + NH3	0.119	0.022	0.023	-0.026	-0.003	0.024	0.065	174
AY9 + Sex + PV1 + NH3	0.102	0.025	0.032	0.008	0.014	0.024	0.078	175
AY5 + Sex + PV1 + NH3	0.102	0.025	0.032	0.009	0.014	0.024	0.079	176
AY9 + PV3 + NH5	0.092	-0.005	-0.006	-0.047	-0.047	-0.045	0.047	177
AY5 + Sex + PV1 + NH1 + AY5:PV1	0.120	0.028	0.010	-0.032	0.004	0.026	0.072	178
PV1 + CD5 + NH1 + CD5:PV1	0.111	-0.011	-0.015	-0.069	-0.037	0.006	0.043	179
AY9 + Sex + PV1 + NH1 + AY9:PV1	0.120	0.028	0.010	-0.031	0.005	0.026	0.072	180
AY5 + PV3 + NH5	0.093	-0.006	-0.007	-0.047	-0.048	-0.045	0.046	181
AY9 + Sex + CD5	0.113	0.043	0.052	-0.022	-0.019	-0.013	0.031	182
AY9 + Sex + PV1 + NH5	0.096	0.027	0.038	0.017	0.012	0.022	0.082	183
PV1 + CD5 + NH1	0.111	-0.012	-0.017	-0.068	-0.037	0.007	0.042	184
AY5 + Sex + PV1 + NH5	0.096	0.027	0.039	0.017	0.012	0.022	0.083	185
PV1 + CD3 + NH1 + CD3:PV1	0.112	-0.014	-0.017	-0.068	-0.037	0.006	0.042	186
AY5 + Sex + CD5	0.116	0.046	0.055	-0.021	-0.017	-0.011	0.033	187
AY9 + Sex + CD3	0.117	0.046	0.057	-0.021	-0.017	-0.011	0.032	188
PV1 + CD3 + NH1	0.115	-0.012	-0.018	-0.070	-0.036	0.008	0.042	189
AY5 + Sex + CD3	0.120	0.049	0.059	-0.020	-0.015	-0.010	0.035	190
AY9 + CD5	0.131	0.058	0.069	-0.003	-0.003	0.000	0.045	191
PV1 + NH3	0.131	0.029	0.033	-0.008	0.006	0.029	0.071	192
Sex + PV1 + CD5 + NH1 + CD5:PV1	0.100	-0.018	-0.025	-0.086	-0.046	0.000	0.036	193
PV1 + NH5	0.123	0.032	0.042	0.005	0.007	0.027	0.077	194
Sex + PV1 + CD5 + NH1	0.101	-0.018	-0.026	-0.085	-0.045	0.002	0.036	195
Sex + PV1 + CD3 + NH1 + CD3:PV1	0.101	-0.020	-0.026	-0.085	-0.045	0.001	0.036	196
PV1 + CD1 + NH1 + CD1:PV1	0.127	-0.003	-0.016	-0.071	-0.033	0.016	0.048	197
PV1 + CD1 + NH1	0.130	0.003	-0.015	-0.073	-0.031	0.017	0.046	198
AY5 + CD5	0.133	0.061	0.071	-0.002	-0.001	0.001	0.047	199
Sex + PV1 + CD3 + NH1	0.104	-0.018	-0.026	-0.086	-0.044	0.004	0.036	200
AY9 + PV3 + CD5 + NH5	0.074	-0.023	-0.018	-0.054	-0.055	-0.053	0.042	201
AY9 + PV3 + CD5 + NH5 + CD5:PV3	0.073	-0.021	-0.016	-0.054	-0.057	-0.052	0.044	202
AY9 + CD3	0.134	0.061	0.073	-0.002	-0.001	0.002	0.046	203
AY9 + PV3 + NH5 + AY9:PV3	0.089	-0.009	-0.011	-0.058	-0.056	-0.056	0.042	204
AY5 + PV3 + CD5 + NH5	0.075	-0.024	-0.019	-0.054	-0.056	-0.053	0.041	205
AY9 + Sex + PV3 + NH5	0.072	-0.017	-0.018	-0.064	-0.059	-0.051	0.041	206
AY5 + PV3 + CD5 + NH5 + CD5:PV3	0.074	-0.023	-0.017	-0.054	-0.058	-0.053	0.044	207
AY9 + Sex + PV1 + NH1	0.125	0.032	0.017	-0.021	0.012	0.038	0.079	208
Sex + PV1 + CD1 + NH1	0.120	-0.002	-0.022	-0.088	-0.037	0.013	0.042	209
AY5 + Sex + PV1 + NH1	0.125	0.033	0.017	-0.021	0.012	0.038	0.080	210
AY5 + PV3 + NH5 + AY5:PV3	0.090	-0.011	-0.012	-0.058	-0.057	-0.056	0.042	211
AY5 + Sex + PV3 + NH5	0.073	-0.019	-0.018	-0.064	-0.060	-0.052	0.040	212
AY5 + CD3	0.137	0.064	0.075	-0.001	0.001	0.003	0.048	213
Sex + PV1 + CD1 + NH1 + CD1:PV1	0.117	-0.008	-0.024	-0.086	-0.040	0.011	0.043	214
AY9 + PV3 + CD3 + NH5 + CD3:PV3	0.069	-0.026	-0.020	-0.057	-0.060	-0.054	0.044	215
AY9 + PV3 + CD3 + NH5	0.071	-0.027	-0.023	-0.059	-0.059	-0.053	0.041	216
AY5 + PV1 + NH3 + AY5:PV1	0.116	0.032	0.040	0.019	0.020	0.024	0.084	217
AY5 + PV3 + CD3 + NH5 + CD3:PV3	0.070	-0.028	-0.021	-0.057	-0.061	-0.054	0.044	218
AY9 + PV3 + NH3	0.092	-0.012	-0.019	-0.066	-0.059	-0.046	0.044	219
AY9 + PV1 + NH3 + AY9:PV1	0.116	0.032	0.040	0.019	0.021	0.025	0.084	220
AY5 + PV3 + CD3 + NH5	0.072	-0.029	-0.024	-0.059	-0.059	-0.053	0.041	221
AY9 + PV1 + CD5 + CD5:PV1	0.102	-0.016	-0.019	-0.076	-0.067	-0.037	0.020	222
AY5 + PV1 + CD5 + CD5:PV1	0.101	-0.016	-0.019	-0.076	-0.068	-0.038	0.020	223
AY9 + PV1 + CD3 + CD3:PV1	0.100	-0.020	-0.022	-0.075	-0.066	-0.037	0.019	224
AY5 + PV1 + CD3 + CD3:PV1	0.100	-0.020	-0.022	-0.075	-0.066	-0.037	0.019	225
AY9 + Sex + CD1	0.136	0.068	0.067	-0.014	-0.011	-0.002	0.042	226
AY5 + PV3 + NH3	0.093	-0.013	-0.019	-0.066	-0.060	-0.046	0.043	227
AY9 + PV1 + CD5	0.102	-0.018	-0.021	-0.077	-0.068	-0.037	0.018	228
AY5 + PV1 + CD5	0.102	-0.017	-0.021	-0.077	-0.068	-0.037	0.018	229

Supplementary Table 12 continued

	Off-season							
	2013	2014	2015	2016	2017	2018	2019	Rank
AY5 + PV1 + CD3	0.101	-0.020	-0.023	-0.078	-0.067	-0.036	0.018	230
AY9 + PV1 + CD3	0.101	-0.020	-0.023	-0.078	-0.066	-0.036	0.018	231
AY9 + PV3 + CD5 + NH5 + AY9:PV3 + CD5:PV3	0.071	-0.025	-0.019	-0.062	-0.063	-0.061	0.041	232
AY5 + PV1 + CD1	0.108	-0.011	-0.032	-0.082	-0.062	-0.031	0.019	233
AY9 + PV1 + CD1	0.108	-0.011	-0.033	-0.082	-0.062	-0.031	0.019	234
AY5 + PV1 + NH1 + AY5:PV1	0.139	0.039	0.023	-0.013	0.014	0.035	0.082	235
AY5 + Sex + CD1	0.140	0.071	0.070	-0.013	-0.008	0.000	0.044	236
AY9 + PV3 + CD5 + NH5 + AY9:PV3	0.072	-0.026	-0.021	-0.064	-0.063	-0.063	0.038	237
AY5 + PV3 + CD5 + NH5 + AY5:PV3 + CD5:PV3	0.071	-0.027	-0.020	-0.062	-0.064	-0.061	0.041	238
AY9 + PV1 + NH1 + AY9:PV1	0.139	0.039	0.023	-0.012	0.015	0.036	0.082	239
AY9 + PV1 + CD5 + AY9:PV1 + CD5:PV1	0.100	-0.018	-0.021	-0.080	-0.069	-0.042	0.019	240
AY9 + PV1 + CD1 + CD1:PV1	0.107	-0.014	-0.033	-0.079	-0.063	-0.033	0.020	241
AY5 + PV1 + CD1 + CD1:PV1	0.106	-0.014	-0.033	-0.080	-0.064	-0.033	0.020	242
AY5 + PV1 + CD5 + AY5:PV1 + CD5:PV1	0.100	-0.018	-0.022	-0.081	-0.070	-0.042	0.019	243
AY5 + PV3 + CD5 + NH5 + AY5:PV3	0.072	-0.028	-0.022	-0.064	-0.064	-0.062	0.038	244
AY9 + Sex + PV3 + CD5 + NH5	0.057	-0.033	-0.029	-0.070	-0.066	-0.059	0.036	245
AY5 + PV1 + NH5 + AY5:PV1	0.110	0.034	0.047	0.029	0.020	0.023	0.089	246
AY9 + PV3 + CD1 + NH5	0.074	-0.024	-0.030	-0.068	-0.062	-0.054	0.039	247
AY9 + PV1 + CD3 + AY9:PV1 + CD3:PV1	0.099	-0.021	-0.024	-0.080	-0.068	-0.042	0.018	248
AY9 + Sex + PV3 + CD5 + NH5 + CD5:PV3	0.056	-0.032	-0.027	-0.071	-0.069	-0.059	0.038	249
AY9 + PV1 + NH5 + AY9:PV1	0.110	0.034	0.047	0.029	0.020	0.023	0.089	250
AY5 + PV1 + CD3 + AY5:PV1 + CD3:PV1	0.098	-0.022	-0.024	-0.080	-0.069	-0.042	0.018	251
AY9 + PV1 + CD5 + AY9:PV1	0.100	-0.019	-0.024	-0.082	-0.070	-0.042	0.016	252
AY5 + Sex + PV3 + CD5 + NH5	0.058	-0.034	-0.030	-0.070	-0.067	-0.059	0.035	253
AY5 + PV3 + CD1 + NH5	0.075	-0.025	-0.031	-0.068	-0.063	-0.054	0.039	254
AY9 + PV3 + CD1 + NH5 + CD1:PV3	0.074	-0.024	-0.029	-0.066	-0.064	-0.056	0.043	255
AY5 + PV1 + CD5 + AY5:PV1	0.100	-0.019	-0.024	-0.082	-0.071	-0.043	0.016	256
AY5 + Sex + PV3 + CD5 + NH5 + CD5:PV3	0.057	-0.033	-0.028	-0.071	-0.070	-0.060	0.037	257
AY9 + PV3 + CD3 + NH5 + AY9:PV3 + CD3:PV3	0.068	-0.030	-0.024	-0.066	-0.066	-0.063	0.041	258
AY9 + PV1 + CD3 + AY9:PV1	0.099	-0.022	-0.026	-0.083	-0.069	-0.042	0.016	259
AY9 + Sex + PV1	0.145	0.027	-0.008	-0.083	-0.050	-0.010	0.034	260
AY9 + PV1	0.164	0.036	0.006	-0.065	-0.041	-0.003	0.042	261
AY5 + Sex + PV1	0.145	0.027	-0.008	-0.083	-0.050	-0.010	0.034	262
AY9 + Sex + PV3 + NH5 + AY9:PV3	0.069	-0.022	-0.024	-0.076	-0.069	-0.064	0.035	263
AY5 + PV1 + CD3 + AY5:PV1	0.099	-0.022	-0.026	-0.084	-0.070	-0.042	0.016	264
AY5 + PV1	0.164	0.036	0.006	-0.065	-0.041	-0.003	0.042	265
AY5 + PV3 + CD1 + NH5 + CD1:PV3	0.075	-0.025	-0.029	-0.066	-0.065	-0.056	0.042	266
AY5 + PV3 + CD3 + NH5 + AY5:PV3 + CD3:PV3	0.068	-0.032	-0.025	-0.066	-0.068	-0.062	0.041	267
AY9 + PV3 + CD3 + NH5 + AY9:PV3	0.068	-0.031	-0.026	-0.069	-0.067	-0.063	0.038	268
AY5 + Sex + PV3 + NH5 + AY5:PV3	0.069	-0.024	-0.025	-0.076	-0.070	-0.064	0.035	269
AY9 + PV1 + AY9:PV1	0.161	0.033	0.001	-0.073	-0.046	-0.012	0.038	270
AY9 + PV3 + CD5 + NH3 + CD5:PV3	0.070	-0.029	-0.029	-0.072	-0.069	-0.054	0.040	271
AY9 + Sex + PV3 + CD3 + NH5 + CD3:PV3	0.053	-0.037	-0.031	-0.073	-0.072	-0.061	0.038	272
AY5 + PV1 + AY5:PV1	0.161	0.033	0.000	-0.073	-0.047	-0.012	0.038	273
AY9 + Sex + PV3 + CD3 + NH5	0.054	-0.037	-0.033	-0.075	-0.070	-0.059	0.036	274
AY9 + PV3 + CD5 + NH3	0.070	-0.031	-0.032	-0.072	-0.067	-0.054	0.038	275
AY9 + PV1 + CD1 + AY9:PV1	0.106	-0.014	-0.036	-0.088	-0.066	-0.039	0.016	276
AY9 + PV1 + CD1 + AY9:PV1 + CD1:PV1	0.105	-0.016	-0.036	-0.085	-0.066	-0.039	0.018	277
Sex + PV1 + NH1	0.152	0.028	0.006	-0.067	-0.010	0.039	0.063	278
AY5 + PV1 + CD1 + AY5:PV1	0.106	-0.014	-0.036	-0.089	-0.067	-0.039	0.016	279
AY5 + PV1 + CD1 + AY5:PV1 + CD1:PV1	0.105	-0.016	-0.036	-0.085	-0.067	-0.039	0.018	280
AY5 + PV3 + CD3 + NH5 + AY5:PV3	0.069	-0.033	-0.027	-0.069	-0.068	-0.063	0.038	281
AY5 + PV3 + CD5 + NH3 + CD5:PV3	0.070	-0.031	-0.030	-0.072	-0.070	-0.054	0.040	282
AY5 + Sex + PV3 + CD3 + NH5	0.055	-0.039	-0.034	-0.075	-0.070	-0.060	0.035	283
AY5 + PV3 + CD5 + NH3	0.071	-0.032	-0.032	-0.072	-0.068	-0.054	0.037	284
AY9 + Sex + PV3 + NH3	0.072	-0.024	-0.030	-0.082	-0.070	-0.053	0.038	285
AY5 + Sex + PV3 + CD3 + NH5 + CD3:PV3	0.053	-0.038	-0.032	-0.073	-0.073	-0.061	0.037	286
AY9 + PV1 + NH3	0.119	0.035	0.044	0.026	0.024	0.032	0.087	287
AY9 + PV3 + NH3 + AY9:PV3	0.089	-0.016	-0.023	-0.076	-0.068	-0.058	0.038	288
AY5 + PV1 + NH3	0.119	0.035	0.044	0.026	0.024	0.032	0.087	289
AY9 + Sex + PV1 + CD5 + CD5:PV1	0.086	-0.025	-0.032	-0.094	-0.077	-0.045	0.011	290
AY5 + Sex + PV1 + CD5 + CD5:PV1	0.086	-0.025	-0.032	-0.094	-0.077	-0.045	0.011	291
AY5 + Sex + PV3 + NH3	0.073	-0.025	-0.030	-0.082	-0.071	-0.053	0.037	292

Supplementary Table 12 continued

	Off-season							
	2013	2014	2015	2016	2017	2018	2019	Rank
AY9 + PV1 + NH1	0.143	0.043	0.028	-0.004	0.021	0.046	0.088	293
AY9 + Sex + PV1 + CD3 + CD3:PV1	0.085	-0.029	-0.035	-0.092	-0.076	-0.045	0.010	294
AY5 + Sex + PV1 + CD3 + CD3:PV1	0.085	-0.029	-0.035	-0.093	-0.076	-0.045	0.010	295
AY5 + Sex + PV1 + CD5	0.087	-0.026	-0.034	-0.095	-0.077	-0.043	0.010	296
AY5 + PV1 + NH1	0.143	0.043	0.028	-0.004	0.021	0.046	0.088	297
AY9 + Sex + PV1 + CD5	0.087	-0.026	-0.034	-0.095	-0.077	-0.043	0.010	298
AY5 + PV3 + NH3 + AY5:PV3	0.089	-0.018	-0.024	-0.076	-0.069	-0.057	0.038	299
AY5 + Sex + PV1 + CD3	0.087	-0.028	-0.036	-0.095	-0.076	-0.042	0.010	300
AY9 + Sex + PV1 + CD3	0.087	-0.028	-0.036	-0.095	-0.076	-0.042	0.010	301
AY9 + PV3 + CD3 + NH3	0.072	-0.032	-0.033	-0.075	-0.069	-0.054	0.038	302
AY9 + PV3 + CD3 + NH3 + CD3:PV3	0.071	-0.031	-0.031	-0.074	-0.072	-0.055	0.040	303
AY9 + Sex + PV1 + AY9:PV1	0.141	0.023	-0.015	-0.092	-0.056	-0.020	0.028	304
AY5 + Sex + PV1 + CD1	0.093	-0.020	-0.044	-0.098	-0.071	-0.037	0.012	305
AY9 + Sex + PV1 + CD1	0.093	-0.020	-0.044	-0.098	-0.070	-0.037	0.012	306
AY5 + Sex + PV1 + AY5:PV1	0.141	0.023	-0.015	-0.093	-0.057	-0.020	0.028	307
AY5 + PV3 + CD3 + NH3	0.073	-0.034	-0.034	-0.075	-0.070	-0.055	0.037	308
PV1 + NH1	0.163	0.035	0.014	-0.051	-0.004	0.043	0.068	309
AY5 + PV3 + CD3 + NH3 + CD3:PV3	0.072	-0.033	-0.031	-0.074	-0.073	-0.056	0.040	310
AY9 + Sex + PV3 + CD5 + NH5 + AY9:PV3 + CD5:PV3	0.054	-0.036	-0.032	-0.080	-0.075	-0.069	0.033	311
AY9 + PV3 + CD1 + NH5 + AY9:PV3	0.071	-0.027	-0.034	-0.077	-0.070	-0.065	0.035	312
AY9 + PV1 + NH5	0.113	0.037	0.052	0.035	0.023	0.029	0.091	313
AY9 + PV3 + CD1 + NH5 + AY9:PV3 + CD1:PV3	0.072	-0.027	-0.032	-0.075	-0.071	-0.065	0.039	314
AY5 + PV1 + NH5	0.113	0.037	0.052	0.035	0.023	0.029	0.092	315
AY5 + Sex + PV1 + CD1 + CD1:PV1	0.092	-0.023	-0.045	-0.097	-0.073	-0.040	0.012	316
AY9 + Sex + PV3 + CD1 + NH5	0.057	-0.035	-0.040	-0.082	-0.073	-0.061	0.034	317
AY9 + Sex + PV1 + CD1 + CD1:PV1	0.092	-0.023	-0.045	-0.097	-0.073	-0.040	0.012	318
AY9 + Sex + PV3 + CD5 + NH5 + AY9:PV3	0.054	-0.037	-0.034	-0.081	-0.075	-0.070	0.031	319
AY5 + Sex + PV3 + CD5 + NH5 + AY5:PV3 + CD5:PV3	0.054	-0.038	-0.033	-0.080	-0.077	-0.069	0.033	320
AY5 + PV3 + CD1 + NH5 + AY5:PV3	0.072	-0.029	-0.035	-0.077	-0.072	-0.064	0.035	321
AY5 + PV3 + CD1 + NH5 + AY5:PV3 + CD1:PV3	0.072	-0.029	-0.033	-0.075	-0.072	-0.065	0.039	322
AY5 + Sex + PV3 + CD1 + NH5	0.058	-0.036	-0.040	-0.082	-0.074	-0.061	0.033	323
AY9 + Sex + PV3 + CD1 + NH5 + CD1:PV3	0.056	-0.035	-0.038	-0.081	-0.075	-0.063	0.037	324
AY9 + Sex + PV1 + CD5 + AY9:PV1 + CD5:PV1	0.084	-0.027	-0.036	-0.100	-0.080	-0.050	0.008	325
AY9 + PV3 + CD5 + NH3 + AY9:PV3 + CD5:PV3	0.067	-0.033	-0.032	-0.080	-0.075	-0.063	0.036	326
AY5 + Sex + PV3 + CD5 + NH5 + AY5:PV3	0.055	-0.039	-0.035	-0.081	-0.076	-0.070	0.031	327
AY5 + Sex + PV1 + CD5 + AY5:PV1 + CD5:PV1	0.084	-0.027	-0.036	-0.100	-0.081	-0.050	0.008	328
AY9 + Sex + PV1 + CD3 + AY9:PV1 + CD3:PV1	0.083	-0.031	-0.038	-0.098	-0.079	-0.050	0.008	329
AY5 + Sex + PV1 + CD3 + AY5:PV1 + CD3:PV1	0.083	-0.031	-0.038	-0.099	-0.080	-0.051	0.008	330
AY5 + Sex + PV3 + CD1 + NH5 + CD1:PV3	0.057	-0.036	-0.039	-0.081	-0.076	-0.063	0.036	331
AY9 + Sex + PV1 + CD5 + AY9:PV1	0.084	-0.029	-0.038	-0.101	-0.081	-0.050	0.006	332
AY9 + PV3 + CD1 + NH3	0.077	-0.028	-0.040	-0.083	-0.072	-0.055	0.035	333
AY5 + PV3 + CD5 + NH3 + AY5:PV3 + CD5:PV3	0.068	-0.035	-0.033	-0.080	-0.076	-0.063	0.036	334
AY5 + Sex + PV1 + CD5 + AY5:PV1	0.084	-0.028	-0.038	-0.101	-0.081	-0.051	0.006	335
AY9 + PV3 + CD5 + NH3 + AY9:PV3	0.068	-0.035	-0.035	-0.081	-0.075	-0.064	0.033	336
AY9 + Sex + PV3 + CD3 + NH5 + AY9:PV3 + CD3:PV3	0.050	-0.041	-0.036	-0.082	-0.079	-0.071	0.033	337
AY9 + Sex + PV1 + CD3 + AY9:PV1	0.084	-0.031	-0.040	-0.102	-0.080	-0.050	0.006	338
AY5 + Sex + PV1 + CD3 + AY5:PV1	0.084	-0.031	-0.040	-0.102	-0.080	-0.050	0.006	339
AY5 + PV3 + CD1 + NH3	0.078	-0.029	-0.040	-0.083	-0.073	-0.056	0.035	340
AY9 + CD1	0.154	0.083	0.083	0.004	0.005	0.010	0.054	341
AY5 + PV3 + CD5 + NH3 + AY5:PV3	0.068	-0.037	-0.035	-0.081	-0.076	-0.064	0.033	342
AY9 + Sex + PV3 + CD5 + NH3	0.053	-0.042	-0.042	-0.088	-0.078	-0.061	0.031	343
AY9 + PV3 + CD1 + NH3 + CD1:PV3	0.076	-0.029	-0.038	-0.081	-0.075	-0.057	0.039	344
AY5 + Sex + PV3 + CD3 + NH5 + AY5:PV3 + CD3:PV3	0.051	-0.043	-0.037	-0.082	-0.080	-0.070	0.033	345
AY9 + Sex + PV3 + CD3 + NH5 + AY9:PV3	0.051	-0.042	-0.038	-0.085	-0.078	-0.071	0.030	346
AY9 + Sex + PV3 + CD5 + NH3 + CD5:PV3	0.052	-0.040	-0.040	-0.088	-0.081	-0.061	0.033	347
AY9 + PV3 + CD3 + NH3 + AY9:PV3 + CD3:PV3	0.069	-0.035	-0.034	-0.082	-0.077	-0.064	0.036	348
AY5 + Sex + PV3 + CD5 + NH3	0.054	-0.043	-0.043	-0.088	-0.079	-0.061	0.031	349
AY9 + Sex + PV1 + CD1 + AY9:PV1	0.090	-0.023	-0.049	-0.107	-0.076	-0.047	0.007	350
AY5 + PV3 + CD1 + NH3 + CD1:PV3	0.077	-0.030	-0.039	-0.081	-0.076	-0.057	0.039	351
AY9 + Sex + PV1 + CD1 + AY9:PV1 + CD1:PV1	0.090	-0.025	-0.049	-0.103	-0.077	-0.047	0.009	352
AY5 + Sex + PV1 + CD1 + AY5:PV1	0.090	-0.023	-0.049	-0.107	-0.077	-0.047	0.007	353
AY5 + Sex + PV3 + CD3 + NH5 + AY5:PV3	0.051	-0.044	-0.039	-0.085	-0.079	-0.071	0.030	354
AY5 + Sex + PV3 + CD5 + NH3 + CD5:PV3	0.053	-0.041	-0.041	-0.089	-0.081	-0.062	0.033	355

	Off-season							
	2013	2014	2015	2016	2017	2018	2019	Rank
AY5 + Sex + PV1 + CD1 + AY5:PV1 + CD1:PV1	0.089	-0.025	-0.049	-0.104	-0.078	-0.047	0.008	356
AY5 + PV3 + CD3 + NH3 + AY5:PV3 + CD3:PV3	0.069	-0.037	-0.035	-0.082	-0.079	-0.064	0.036	357
AY9 + PV3 + CD3 + NH3 + AY9:PV3	0.069	-0.036	-0.037	-0.085	-0.077	-0.065	0.033	358
AY9 + Sex + PV3 + CD3 + NH3	0.055	-0.042	-0.043	-0.090	-0.080	-0.061	0.032	359
AY9 + Sex + PV3 + NH3 + AY9:PV3	0.068	-0.029	-0.036	-0.094	-0.080	-0.066	0.030	360
AY9 + Sex + PV3 + CD3 + NH3 + CD3:PV3	0.054	-0.042	-0.041	-0.090	-0.083	-0.063	0.034	361
AY5 + PV3 + CD3 + NH3 + AY5:PV3	0.070	-0.038	-0.038	-0.085	-0.079	-0.065	0.033	362
AY5 + Sex + PV3 + CD3 + NH3	0.056	-0.044	-0.044	-0.091	-0.081	-0.061	0.031	363
AY5 + Sex + PV3 + NH3 + AY5:PV3	0.068	-0.031	-0.037	-0.094	-0.082	-0.066	0.030	364
AY5 + CD1	0.157	0.086	0.086	0.005	0.007	0.012	0.057	365
AY5 + Sex + PV3 + CD3 + NH3 + CD3:PV3	0.054	-0.043	-0.042	-0.090	-0.084	-0.063	0.033	366
AY9 + Sex + PV3 + CD1 + NH5 + AY9:PV3	0.053	-0.039	-0.045	-0.093	-0.082	-0.073	0.028	367
AY9 + Sex + PV3 + CD1 + NH5 + AY9:PV3 + CD1:PV3	0.053	-0.039	-0.043	-0.090	-0.082	-0.073	0.032	368
AY5 + Sex + PV3 + CD1 + NH5 + AY5:PV3	0.054	-0.041	-0.046	-0.093	-0.083	-0.072	0.028	369
AY5 + Sex + PV3 + CD1 + NH5 + AY5:PV3 + CD1:PV3	0.054	-0.041	-0.044	-0.091	-0.084	-0.073	0.032	370
AY9 + Sex + PV3 + CD1 + NH3	0.059	-0.039	-0.049	-0.097	-0.083	-0.062	0.030	371
AY9 + PV3 + CD1 + NH3 + AY9:PV3	0.074	-0.032	-0.043	-0.092	-0.081	-0.067	0.030	372
AY9 + PV3 + CD1 + NH3 + AY9:PV3 + CD1:PV3	0.074	-0.032	-0.041	-0.090	-0.081	-0.066	0.035	373
AY5 + Sex + PV3 + CD1 + NH3	0.060	-0.040	-0.049	-0.097	-0.084	-0.062	0.030	374
AY9 + Sex + PV3 + CD5 + NH3 + AY9:PV3 + CD5:PV3	0.050	-0.044	-0.044	-0.097	-0.087	-0.072	0.028	375
AY5 + PV3 + CD1 + NH3 + AY5:PV3	0.074	-0.034	-0.044	-0.093	-0.082	-0.066	0.030	376
AY5 + PV3 + CD1 + NH3 + AY5:PV3 + CD1:PV3	0.074	-0.034	-0.042	-0.090	-0.082	-0.066	0.035	377
AY9 + Sex + PV3 + CD1 + NH3 + CD1:PV3	0.058	-0.040	-0.047	-0.096	-0.085	-0.064	0.034	378
AY9 + Sex + PV3 + CD5 + NH3 + AY9:PV3	0.050	-0.046	-0.047	-0.098	-0.087	-0.073	0.025	379
AY5 + Sex + PV3 + CD5 + NH3 + AY5:PV3 + CD5:PV3	0.050	-0.046	-0.045	-0.097	-0.088	-0.071	0.028	380
AY5 + Sex + PV3 + CD1 + NH3 + CD1:PV3	0.059	-0.041	-0.048	-0.096	-0.086	-0.064	0.033	381
AY5 + Sex + PV3 + CD5 + NH3 + AY5:PV3	0.051	-0.048	-0.048	-0.098	-0.088	-0.072	0.025	382
AY9 + Sex + PV3 + CD3 + NH3 + AY9:PV3 + CD3:PV3	0.051	-0.046	-0.046	-0.098	-0.090	-0.073	0.029	383
AY9 + Sex + PV3 + CD3 + NH3 + AY9:PV3	0.052	-0.047	-0.048	-0.101	-0.089	-0.074	0.025	384
AY5 + Sex + PV3 + CD3 + NH3 + AY5:PV3 + CD3:PV3	0.051	-0.048	-0.047	-0.099	-0.091	-0.073	0.028	385
AY5 + Sex + PV3 + CD3 + NH3 + AY5:PV3	0.052	-0.049	-0.049	-0.101	-0.090	-0.073	0.025	386
CD5	0.179	0.090	0.088	-0.022	-0.023	-0.003	0.039	387
Sex + CD5	0.173	0.085	0.082	-0.033	-0.031	-0.007	0.035	388
AY9 + Sex + PV3 + CD1 + NH3 + AY9:PV3	0.055	-0.043	-0.054	-0.108	-0.092	-0.075	0.023	389
AY9 + Sex + PV3 + CD1 + NH3 + AY9:PV3 + CD1:PV3	0.055	-0.044	-0.052	-0.106	-0.093	-0.075	0.028	390
AY9 + Sex + CD5 + NH1	0.129	0.067	0.082	0.024	0.033	0.037	0.081	391
AY5 + Sex + PV3 + CD1 + NH3 + AY5:PV3	0.056	-0.045	-0.055	-0.108	-0.093	-0.075	0.023	392
AY5 + Sex + PV3 + CD1 + NH3 + AY5:PV3 + CD1:PV3	0.056	-0.046	-0.053	-0.106	-0.094	-0.075	0.028	393
CD3	0.186	0.095	0.095	-0.020	-0.020	0.000	0.041	394
Sex + CD3	0.180	0.090	0.089	-0.030	-0.027	-0.004	0.038	395
AY9 + PV3 + NH1	0.137	-0.002	-0.040	-0.112	-0.090	-0.056	0.027	396
AY5 + Sex + CD5 + NH1	0.131	0.070	0.084	0.025	0.035	0.038	0.083	397
AY5 + PV3 + NH1	0.138	-0.004	-0.041	-0.113	-0.091	-0.056	0.026	398
PV3 + NH5	0.108	-0.039	-0.044	-0.100	-0.095	-0.075	0.011	399
AY9 + PV3 + CD5 + NH1 + CD5:PV3	0.096	-0.029	-0.053	-0.113	-0.096	-0.065	0.024	400
AY9 + PV3 + CD5 + NH1	0.097	-0.031	-0.055	-0.113	-0.095	-0.065	0.021	401
AY9 + Sex + CD3 + NH1	0.134	0.071	0.087	0.026	0.036	0.040	0.083	402
AY5 + PV3 + CD5 + NH1 + CD5:PV3	0.097	-0.030	-0.053	-0.113	-0.097	-0.065	0.023	403
AY5 + PV3 + CD5 + NH1	0.098	-0.032	-0.056	-0.114	-0.096	-0.065	0.021	404
PV3 + CD5 + NH5	0.086	-0.055	-0.054	-0.104	-0.100	-0.081	0.008	405
AY5 + Sex + CD3 + NH1	0.136	0.074	0.089	0.027	0.038	0.041	0.086	406
PV3 + CD5 + NH5 + CD5:PV3	0.084	-0.054	-0.054	-0.105	-0.103	-0.081	0.009	407
AY9 + PV3 + CD3 + NH1 + CD3:PV3	0.094	-0.034	-0.056	-0.116	-0.101	-0.067	0.023	408
AY9 + Sex + PV3 + NH1	0.116	-0.015	-0.050	-0.128	-0.100	-0.063	0.022	409
AY9 + PV3 + CD3 + NH1	0.096	-0.035	-0.059	-0.118	-0.099	-0.066	0.021	410
AY9 + PV3 + NH1 + AY9:PV3	0.132	-0.006	-0.045	-0.123	-0.099	-0.068	0.021	411
AY9 + PV3 + CD5 + NH1 + AY9:PV3 + CD5:PV3	0.093	-0.032	-0.056	-0.120	-0.102	-0.074	0.020	412
AY5 + PV3 + CD3 + NH1 + CD3:PV3	0.095	-0.036	-0.057	-0.116	-0.102	-0.067	0.022	413
AY5 + PV3 + CD3 + NH1	0.097	-0.036	-0.059	-0.118	-0.100	-0.066	0.020	414
AY5 + Sex + PV3 + NH1	0.117	-0.016	-0.051	-0.128	-0.101	-0.063	0.021	415
AY5 + PV3 + NH1 + AY5:PV3	0.132	-0.008	-0.047	-0.123	-0.101	-0.068	0.021	416
AY9 + PV3 + CD5 + NH1 + AY9:PV3	0.094	-0.033	-0.058	-0.122	-0.102	-0.075	0.017	417
AY5 + PV3 + CD5 + NH1 + AY5:PV3 + CD5:PV3	0.094	-0.034	-0.057	-0.120	-0.103	-0.073	0.020	418

Supplementary Table 12 continued

	Off-season							
	2013	2014	2015	2016	2017	2018	2019	Rank
AY5 + PV3 + CD5 + NH1 + AY5:PV3	0.094	-0.036	-0.059	-0.122	-0.103	-0.075	0.017	419
PV3 + CD3 + NH5	0.083	-0.060	-0.060	-0.110	-0.105	-0.082	0.007	420
AY9 + Sex + PV3 + CD5 + NH1 + CD5:PV3	0.079	-0.040	-0.063	-0.129	-0.107	-0.072	0.017	421
AY9 + Sex + PV3 + CD5 + NH1	0.080	-0.042	-0.065	-0.129	-0.105	-0.072	0.016	422
PV3 + CD3 + NH5 + CD3:PV3	0.081	-0.060	-0.059	-0.109	-0.108	-0.084	0.008	423
AY9 + PV3 + CD1 + NH1	0.109	-0.025	-0.061	-0.127	-0.104	-0.068	0.018	424
AY5 + Sex + PV3 + CD5 + NH1 + CD5:PV3	0.080	-0.041	-0.064	-0.129	-0.108	-0.073	0.017	425
AY5 + Sex + PV3 + CD5 + NH1	0.082	-0.043	-0.065	-0.129	-0.106	-0.072	0.015	426
AY9 + PV3 + CD3 + NH1 + AY9:PV3 + CD3:PV3	0.092	-0.037	-0.059	-0.123	-0.107	-0.076	0.019	427
PV1	0.201	0.019	-0.032	-0.160	-0.087	-0.012	0.004	428
AY5 + PV3 + CD1 + NH1	0.110	-0.027	-0.062	-0.127	-0.105	-0.068	0.017	429
AY9 + PV3 + CD3 + NH1 + AY9:PV3	0.093	-0.038	-0.062	-0.127	-0.106	-0.077	0.016	430
AY5 + PV3 + CD3 + NH1 + AY5:PV3 + CD3:PV3	0.092	-0.039	-0.061	-0.124	-0.108	-0.076	0.019	431
AY9 + PV3 + CD1 + NH1 + CD1:PV3	0.108	-0.027	-0.060	-0.126	-0.108	-0.070	0.021	432
AY9 + Sex + PV3 + CD3 + NH1	0.079	-0.045	-0.068	-0.133	-0.109	-0.072	0.015	433
Sex + PV1	0.190	0.016	-0.039	-0.172	-0.090	-0.013	0.003	434
AY9 + Sex + PV3 + CD3 + NH1 + CD3:PV3	0.077	-0.045	-0.066	-0.131	-0.112	-0.074	0.017	435
AY5 + PV3 + CD3 + NH1 + AY5:PV3	0.093	-0.040	-0.063	-0.127	-0.108	-0.076	0.016	436
AY5 + Sex + PV3 + CD3 + NH1	0.080	-0.047	-0.069	-0.133	-0.110	-0.073	0.015	437
AY5 + PV3 + CD1 + NH1 + CD1:PV3	0.109	-0.029	-0.061	-0.126	-0.109	-0.071	0.020	438
AY5 + Sex + PV3 + CD3 + NH1 + CD3:PV3	0.078	-0.046	-0.067	-0.132	-0.113	-0.075	0.016	439
AY9 + Sex + PV3 + NH1 + AY9:PV3	0.110	-0.020	-0.057	-0.139	-0.110	-0.077	0.014	440
Sex + PV3 + NH5	0.093	-0.053	-0.059	-0.123	-0.112	-0.085	0.003	441
AY9 + Sex + PV3 + CD5 + NH1 + AY9:PV3 + CD5:PV3	0.076	-0.044	-0.067	-0.137	-0.113	-0.082	0.013	442
AY5 + Sex + PV3 + NH1 + AY5:PV3	0.111	-0.022	-0.058	-0.140	-0.112	-0.077	0.014	443
PV3 + CD1 + NH5	0.089	-0.058	-0.068	-0.121	-0.112	-0.085	0.003	444
AY9 + Sex + PV3 + CD5 + NH1 + AY9:PV3	0.077	-0.045	-0.069	-0.139	-0.113	-0.083	0.010	445
AY5 + Sex + PV3 + CD5 + NH1 + AY5:PV3 + CD5:PV3	0.077	-0.046	-0.068	-0.137	-0.115	-0.082	0.012	446
AY9 + Sex + PV3 + CD1 + NH1	0.091	-0.036	-0.070	-0.141	-0.114	-0.074	0.013	447
AY9 + PV3 + CD1 + NH1 + AY9:PV3	0.104	-0.029	-0.065	-0.136	-0.113	-0.080	0.012	448
AY5 + Sex + PV3 + CD5 + NH1 + AY5:PV3	0.077	-0.047	-0.070	-0.139	-0.115	-0.083	0.010	449
PV3 + NH3	0.109	-0.049	-0.061	-0.126	-0.114	-0.080	0.002	450
AY9 + PV3 + CD1 + NH1 + AY9:PV3 + CD1:PV3	0.105	-0.029	-0.064	-0.134	-0.114	-0.080	0.017	451
PV3 + CD1 + NH5 + CD1:PV3	0.088	-0.059	-0.068	-0.121	-0.115	-0.088	0.005	452
AY5 + Sex + PV3 + CD1 + NH1	0.093	-0.038	-0.070	-0.141	-0.115	-0.075	0.012	453
AY5 + PV3 + CD1 + NH1 + AY5:PV3	0.105	-0.031	-0.066	-0.136	-0.114	-0.080	0.012	454
Sex + PV3 + CD5 + NH5	0.074	-0.068	-0.069	-0.126	-0.117	-0.091	-0.001	455
AY9 + Sex + PV3 + CD3 + NH1 + AY9:PV3 + CD3:PV3	0.074	-0.048	-0.071	-0.140	-0.118	-0.084	0.012	456
AY5 + PV3 + CD1 + NH1 + AY5:PV3 + CD1:PV3	0.105	-0.032	-0.065	-0.134	-0.115	-0.080	0.017	457
Sex + PV3 + CD5 + NH5 + CD5:PV3	0.072	-0.067	-0.069	-0.128	-0.120	-0.092	0.000	458
AY9 + Sex + PV3 + CD1 + NH1 + CD1:PV3	0.091	-0.038	-0.069	-0.141	-0.118	-0.077	0.016	459
AY9 + Sex + PV3 + CD3 + NH1 + AY9:PV3	0.075	-0.049	-0.073	-0.143	-0.118	-0.085	0.009	460
PV3 + CD5 + NH3 + CD5:PV3	0.080	-0.066	-0.071	-0.128	-0.120	-0.087	0.000	461
AY5 + Sex + PV3 + CD3 + NH1 + AY5:PV3 + CD3:PV3	0.075	-0.050	-0.072	-0.140	-0.120	-0.084	0.012	462
PV3 + CD5 + NH3	0.082	-0.067	-0.072	-0.127	-0.118	-0.086	-0.001	463
Sex + CD1	0.213	0.126	0.113	-0.016	-0.013	0.015	0.059	464
AY5 + Sex + PV3 + CD1 + NH1 + CD1:PV3	0.092	-0.040	-0.070	-0.141	-0.119	-0.078	0.015	465
AY5 + Sex + PV3 + CD3 + NH1 + AY5:PV3	0.076	-0.051	-0.074	-0.143	-0.119	-0.084	0.009	466
AY9 + CD5 + NH1	0.146	0.082	0.097	0.042	0.048	0.049	0.094	467
CD1	0.219	0.130	0.118	-0.008	-0.007	0.018	0.060	468
Sex + PV3 + CD3 + NH5	0.071	-0.073	-0.074	-0.132	-0.121	-0.092	-0.002	469
Sex + PV3 + CD3 + NH5 + CD3:PV3	0.068	-0.073	-0.074	-0.132	-0.125	-0.094	-0.001	470
PV1 + CD5	0.115	-0.043	-0.060	-0.157	-0.115	-0.056	-0.023	471
AY5 + CD5 + NH1	0.148	0.084	0.099	0.042	0.050	0.050	0.096	472
AY9 + Sex + PV3 + CD1 + NH1 + AY9:PV3	0.087	-0.041	-0.076	-0.151	-0.123	-0.088	0.006	473
PV1 + CD5 + CD5:PV1	0.114	-0.043	-0.060	-0.158	-0.116	-0.058	-0.024	474
PV1 + CD3	0.115	-0.045	-0.062	-0.158	-0.114	-0.055	-0.024	475
PV3 + CD3 + NH3	0.085	-0.069	-0.075	-0.133	-0.122	-0.088	-0.003	476
AY9 + Sex + PV3 + CD1 + NH1 + AY9:PV3 + CD1:PV3	0.086	-0.041	-0.074	-0.149	-0.124	-0.088	0.011	477
Sex + CD5 + NH1	0.175	0.101	0.109	0.022	0.030	0.047	0.091	478
PV3 + CD3 + NH3 + CD3:PV3	0.083	-0.069	-0.074	-0.133	-0.126	-0.090	-0.002	479
AY5 + Sex + PV3 + CD1 + NH1 + AY5:PV3	0.087	-0.043	-0.077	-0.152	-0.125	-0.088	0.006	480
AY5 + Sex + PV3 + CD1 + NH1 + AY5:PV3 + CD1:PV3	0.087	-0.044	-0.075	-0.150	-0.126	-0.088	0.010	481

Supplementary Table 12 continued

	Off-season							
	2013	2014	2015	2016	2017	2018	2019	Rank
AY9 + Sex + CD5 + NH3	0.127	0.072	0.099	0.056	0.063	0.058	0.105	482
PV1 + CD3 + CD3:PV1	0.113	-0.047	-0.063	-0.158	-0.116	-0.058	-0.025	483
AY9 + Sex + CD1 + NH1	0.154	0.094	0.103	0.036	0.047	0.052	0.095	484
AY9 + CD3 + NH1	0.151	0.086	0.102	0.044	0.051	0.052	0.096	485
PV1 + CD1	0.128	-0.035	-0.072	-0.167	-0.112	-0.049	-0.023	486
AY5 + Sex + CD5 + NH3	0.130	0.074	0.101	0.056	0.065	0.059	0.107	487
AY5 + CD3 + NH1	0.153	0.088	0.104	0.044	0.053	0.053	0.098	488
AY5 + Sex + CD1 + NH1	0.157	0.097	0.105	0.037	0.049	0.053	0.098	489
Sex + PV3 + CD1 + NH5	0.076	-0.071	-0.082	-0.142	-0.127	-0.094	-0.005	490
Sex + PV3 + CD1 + NH5 + CD1:PV3	0.074	-0.072	-0.082	-0.142	-0.131	-0.097	-0.004	491
Sex + PV1 + CD5	0.105	-0.048	-0.070	-0.172	-0.122	-0.059	-0.028	492
AY9 + Sex + CD3 + NH5	0.122	0.071	0.103	0.063	0.070	0.062	0.113	493
PV1 + CD1 + CD1:PV1	0.125	-0.041	-0.075	-0.168	-0.116	-0.053	-0.026	494
Sex + PV1 + CD3	0.106	-0.049	-0.071	-0.173	-0.121	-0.057	-0.028	495
Sex + PV3 + NH3	0.094	-0.064	-0.076	-0.148	-0.130	-0.090	-0.006	496
AY9 + Sex + CD5 + NH5	0.121	0.071	0.102	0.064	0.071	0.063	0.115	497
Sex + PV1 + CD5 + CD5:PV1	0.104	-0.048	-0.069	-0.173	-0.124	-0.061	-0.029	498
PV3 + CD1 + NH3	0.092	-0.066	-0.082	-0.143	-0.128	-0.090	-0.007	499
Sex + PV1 + CD1	0.119	-0.039	-0.079	-0.181	-0.117	-0.051	-0.026	500
AY9 + Sex + CD1 + NH5	0.127	0.079	0.104	0.061	0.069	0.061	0.113	501
PV3 + CD1 + NH3 + CD1:PV3	0.091	-0.068	-0.083	-0.144	-0.132	-0.093	-0.004	502
AY5 + Sex + CD3 + NH5	0.124	0.073	0.105	0.063	0.072	0.063	0.115	503
Sex + PV1 + CD3 + CD3:PV1	0.104	-0.052	-0.072	-0.173	-0.123	-0.062	-0.030	504
AY9 + Sex + CD3 + NH3	0.134	0.078	0.106	0.060	0.068	0.062	0.109	505
AY5 + Sex + CD5 + NH5	0.124	0.073	0.104	0.065	0.072	0.064	0.117	506
Sex + CD3 + NH1	0.184	0.108	0.118	0.027	0.036	0.052	0.096	507
Sex + PV3 + CD5 + NH3	0.069	-0.080	-0.086	-0.149	-0.134	-0.096	-0.010	508
AY5 + Sex + CD1 + NH5	0.130	0.081	0.106	0.061	0.070	0.062	0.115	509
Sex + PV3 + CD5 + NH3 + CD5:PV3	0.067	-0.079	-0.086	-0.151	-0.137	-0.097	-0.009	510
CD5 + NH1	0.182	0.107	0.116	0.034	0.040	0.053	0.096	511
AY5 + Sex + CD3 + NH3	0.136	0.080	0.108	0.060	0.070	0.063	0.111	512
Sex + PV1 + CD1 + CD1:PV1	0.116	-0.046	-0.082	-0.182	-0.122	-0.056	-0.029	513
AY9 + Sex + CD1 + NH3	0.141	0.087	0.109	0.060	0.069	0.064	0.111	514
Sex + PV3 + CD3 + NH3	0.072	-0.082	-0.088	-0.154	-0.137	-0.097	-0.011	515
Sex + PV3 + CD3 + NH3 + CD3:PV3	0.070	-0.082	-0.088	-0.154	-0.142	-0.100	-0.011	516
AY5 + Sex + CD1 + NH3	0.143	0.090	0.111	0.061	0.071	0.065	0.113	517
AY9 + Sex	0.202	0.131	0.127	0.033	0.040	0.049	0.086	518
CD3 + NH1	0.191	0.114	0.124	0.038	0.044	0.058	0.100	519
Sex + PV3 + CD1 + NH3	0.079	-0.079	-0.095	-0.163	-0.143	-0.100	-0.014	520
Sex + PV3 + CD1 + NH3 + CD1:PV3	0.077	-0.081	-0.095	-0.164	-0.147	-0.103	-0.013	521
AY9 + CD1 + NH1	0.172	0.109	0.118	0.053	0.061	0.064	0.107	522
AY9 + CD5 + NH3	0.144	0.086	0.114	0.074	0.079	0.070	0.118	523
Sex + CD5 + NH3	0.166	0.101	0.122	0.056	0.063	0.068	0.115	524
AY5 + Sex	0.207	0.135	0.131	0.035	0.043	0.051	0.089	525
AY5 + CD5 + NH3	0.146	0.088	0.116	0.074	0.080	0.071	0.120	526
AY5 + CD1 + NH1	0.174	0.112	0.120	0.054	0.063	0.065	0.110	527
Sex + CD5 + NH5	0.157	0.099	0.124	0.064	0.071	0.072	0.124	528
AY9 + CD3 + NH5	0.139	0.086	0.119	0.081	0.086	0.074	0.126	529
AY9 + CD5 + NH5	0.138	0.086	0.117	0.083	0.086	0.075	0.128	530
Sex + CD3 + NH5	0.159	0.100	0.126	0.064	0.071	0.072	0.123	531
AY5 + CD3 + NH5	0.141	0.088	0.120	0.082	0.087	0.075	0.128	532
AY9 + CD1 + NH5	0.146	0.094	0.119	0.079	0.084	0.074	0.126	533
AY9 + CD3 + NH3	0.151	0.092	0.121	0.077	0.084	0.074	0.122	534
AY5 + CD5 + NH5	0.140	0.088	0.119	0.083	0.088	0.076	0.130	535
AY5 + CD1 + NH5	0.148	0.096	0.121	0.079	0.085	0.074	0.127	536
AY5 + CD3 + NH3	0.153	0.094	0.123	0.078	0.085	0.075	0.124	537
Sex + CD1 + NH5	0.168	0.111	0.130	0.064	0.071	0.074	0.125	538
AY9 + PV3 + CD5 + CD5:PV3	0.096	-0.052	-0.085	-0.175	-0.167	-0.136	-0.034	539
Sex + CD3 + NH3	0.176	0.110	0.132	0.062	0.071	0.075	0.121	540
AY9 + CD1 + NH3	0.159	0.103	0.124	0.078	0.084	0.076	0.123	541
AY9 + PV3 + CD5	0.097	-0.054	-0.086	-0.175	-0.166	-0.136	-0.036	542
CD5 + NH3	0.173	0.107	0.130	0.069	0.074	0.075	0.121	543
AY5 + PV3 + CD5 + CD5:PV3	0.097	-0.054	-0.086	-0.175	-0.169	-0.136	-0.035	544

Supplementary Table 12 continued

	Off-season							
	2013	2014	2015	2016	2017	2018	2019	Rank
AY5 + PV3 + CD5	0.099	-0.055	-0.087	-0.176	-0.167	-0.136	-0.037	545
AY5 + CD1 + NH3	0.161	0.105	0.126	0.078	0.086	0.077	0.125	546
AY9 + PV3 + CD3 + CD3:PV3	0.090	-0.059	-0.091	-0.179	-0.172	-0.137	-0.034	547
AY9 + PV3 + CD3	0.092	-0.060	-0.092	-0.181	-0.170	-0.135	-0.035	548
AY5 + PV3 + CD3 + CD3:PV3	0.092	-0.061	-0.092	-0.179	-0.174	-0.137	-0.035	549
AY5 + PV3 + CD3	0.094	-0.061	-0.093	-0.181	-0.171	-0.136	-0.036	550
AY9 + PV3 + CD5 + AY9:PV3 + CD5:PV3	0.093	-0.055	-0.088	-0.183	-0.173	-0.144	-0.037	551
CD5 + NH5	0.165	0.105	0.132	0.079	0.082	0.080	0.131	552
CD3 + NH5	0.167	0.106	0.134	0.078	0.083	0.079	0.130	553
AY9 + PV3 + CD5 + AY9:PV3	0.094	-0.056	-0.089	-0.185	-0.173	-0.145	-0.039	554
AY5 + PV3 + CD5 + AY5:PV3 + CD5:PV3	0.094	-0.058	-0.089	-0.184	-0.175	-0.144	-0.038	555
PV3 + CD5 + NH1 + CD5:PV3	0.114	-0.076	-0.107	-0.188	-0.165	-0.109	-0.028	556
PV3 + CD5 + NH1	0.116	-0.077	-0.108	-0.187	-0.163	-0.108	-0.029	557
AY5 + PV3 + CD5 + AY5:PV3	0.095	-0.059	-0.091	-0.185	-0.175	-0.145	-0.040	558
AY9 + PV3 + CD3 + AY9:PV3 + CD3:PV3	0.088	-0.062	-0.094	-0.188	-0.178	-0.145	-0.037	559
AY9	0.224	0.149	0.146	0.054	0.058	0.063	0.100	560
AY9 + Sex + NH5	0.151	0.104	0.132	0.089	0.098	0.088	0.135	561
AY9 + PV3 + CD3 + AY9:PV3	0.089	-0.062	-0.095	-0.190	-0.177	-0.145	-0.039	562
AY9 + Sex + PV3 + CD5 + CD5:PV3	0.079	-0.063	-0.098	-0.193	-0.180	-0.143	-0.042	563
AY5 + PV3 + CD3 + AY5:PV3 + CD3:PV3	0.088	-0.064	-0.095	-0.188	-0.179	-0.145	-0.038	564
AY9 + Sex + PV3 + CD5	0.081	-0.065	-0.099	-0.193	-0.178	-0.142	-0.043	565
AY9 + Sex + NH1	0.188	0.128	0.136	0.066	0.080	0.082	0.120	566
Sex + CD1 + NH1	0.217	0.145	0.147	0.045	0.055	0.074	0.118	567
Sex + CD1 + NH3	0.188	0.125	0.141	0.065	0.075	0.081	0.127	568
CD3 + NH3	0.183	0.116	0.140	0.075	0.081	0.081	0.127	569
CD1 + NH5	0.176	0.118	0.138	0.077	0.082	0.081	0.131	570
AY5 + PV3 + CD3 + AY5:PV3	0.090	-0.065	-0.096	-0.190	-0.179	-0.145	-0.040	571
AY5 + Sex + PV3 + CD5 + CD5:PV3	0.080	-0.065	-0.099	-0.193	-0.181	-0.144	-0.043	572
AY9 + PV3 + CD1	0.104	-0.052	-0.103	-0.193	-0.177	-0.138	-0.040	573
AY5 + Sex + PV3 + CD5	0.082	-0.066	-0.100	-0.193	-0.179	-0.143	-0.043	574
AY5 + Sex + NH5	0.154	0.106	0.134	0.090	0.100	0.090	0.137	575
AY9 + Sex + PV3 + CD3 + CD3:PV3	0.074	-0.070	-0.103	-0.196	-0.185	-0.144	-0.041	576
AY9 + Sex + PV3 + CD3	0.076	-0.070	-0.104	-0.197	-0.182	-0.142	-0.042	577
AY9 + PV3 + CD1 + CD1:PV3	0.103	-0.054	-0.103	-0.193	-0.181	-0.141	-0.039	578
AY9 + Sex + NH3	0.163	0.111	0.135	0.086	0.097	0.089	0.131	579
AY5 + PV3 + CD1	0.105	-0.054	-0.104	-0.193	-0.178	-0.139	-0.041	580
AY5	0.228	0.153	0.150	0.056	0.061	0.065	0.103	581
AY9 + PV3	0.158	-0.019	-0.079	-0.195	-0.181	-0.142	-0.043	582
AY5 + Sex + NH1	0.191	0.132	0.139	0.067	0.082	0.084	0.123	583
AY5 + Sex + PV3 + CD3	0.077	-0.072	-0.105	-0.197	-0.183	-0.142	-0.043	584
AY5 + Sex + PV3 + CD3 + CD3:PV3	0.075	-0.071	-0.104	-0.196	-0.186	-0.144	-0.042	585
PV3 + CD3 + NH1	0.117	-0.083	-0.114	-0.195	-0.170	-0.111	-0.032	586
AY5 + PV3 + CD1 + CD1:PV3	0.104	-0.056	-0.104	-0.193	-0.182	-0.142	-0.040	587
PV3 + CD3 + NH1 + CD3:PV3	0.113	-0.083	-0.113	-0.194	-0.174	-0.113	-0.031	588
AY5 + Sex + NH3	0.166	0.114	0.138	0.087	0.099	0.090	0.134	589
AY5 + PV3	0.160	-0.021	-0.081	-0.196	-0.183	-0.143	-0.044	590
PV3 + NH1	0.169	-0.055	-0.100	-0.199	-0.170	-0.107	-0.030	591
AY9 + Sex + PV3 + CD5 + AY9:PV3 + CD5:PV3	0.076	-0.067	-0.102	-0.202	-0.186	-0.152	-0.046	592
AY9 + Sex + PV3 + CD5 + AY9:PV3	0.077	-0.068	-0.103	-0.203	-0.186	-0.153	-0.047	593
AY5 + Sex + PV3 + CD5 + AY5:PV3 + CD5:PV3	0.077	-0.070	-0.103	-0.202	-0.188	-0.152	-0.046	594
AY9 + PV3 + CD1 + AY9:PV3 + CD1:PV3	0.099	-0.056	-0.106	-0.201	-0.186	-0.150	-0.042	595
AY9 + PV3 + CD1 + AY9:PV3	0.099	-0.055	-0.106	-0.202	-0.184	-0.148	-0.044	596
CD1 + NH1	0.224	0.150	0.151	0.054	0.061	0.078	0.121	597
AY5 + Sex + PV3 + CD5 + AY5:PV3	0.078	-0.070	-0.105	-0.203	-0.187	-0.153	-0.048	598
AY9 + Sex + PV3 + CD3 + AY9:PV3 + CD3:PV3	0.071	-0.073	-0.107	-0.205	-0.191	-0.153	-0.045	599
AY5 + PV3 + CD1 + AY5:PV3	0.100	-0.058	-0.108	-0.202	-0.186	-0.148	-0.045	600
AY5 + PV3 + CD1 + AY5:PV3 + CD1:PV3	0.099	-0.059	-0.107	-0.202	-0.188	-0.150	-0.043	601
AY9 + Sex + PV3 + CD3 + AY9:PV3	0.072	-0.073	-0.108	-0.207	-0.190	-0.153	-0.047	602
Sex + PV3 + CD5 + NH1	0.104	-0.089	-0.120	-0.207	-0.177	-0.117	-0.036	603
AY9 + Sex + PV3 + CD1	0.087	-0.064	-0.113	-0.208	-0.188	-0.145	-0.046	604
Sex + PV3 + CD5 + NH1 + CD5:PV3	0.102	-0.089	-0.120	-0.208	-0.180	-0.118	-0.036	605
AY5 + Sex + PV3 + CD3 + AY5:PV3 + CD3:PV3	0.072	-0.076	-0.108	-0.206	-0.192	-0.153	-0.046	606
AY9 + PV3 + AY9:PV3	0.152	-0.022	-0.084	-0.206	-0.190	-0.153	-0.047	607

Supplementary Table 12 continued

	Off-season							
	2013	2014	2015	2016	2017	2018	2019	Rank
CD1 + NH3	0.195	0.131	0.147	0.077	0.084	0.086	0.132	608
AY5 + Sex + PV3 + CD3 + AY5:PV3	0.073	-0.076	-0.110	-0.208	-0.191	-0.153	-0.047	609
AY9 + Sex + PV3	0.136	-0.031	-0.092	-0.212	-0.193	-0.148	-0.048	610
AY5 + Sex + PV3 + CD1	0.088	-0.065	-0.114	-0.209	-0.190	-0.145	-0.047	611
AY9 + Sex + PV3 + CD1 + CD1:PV3	0.085	-0.065	-0.114	-0.209	-0.193	-0.148	-0.045	612
AY5 + PV3 + AY5:PV3	0.153	-0.025	-0.086	-0.207	-0.192	-0.153	-0.048	613
AY5 + Sex + PV3 + CD1 + CD1:PV3	0.087	-0.067	-0.115	-0.210	-0.194	-0.149	-0.046	614
AY5 + Sex + PV3	0.138	-0.033	-0.093	-0.212	-0.194	-0.149	-0.049	615
Sex + PV3 + CD3 + NH1	0.105	-0.095	-0.125	-0.214	-0.183	-0.119	-0.039	616
PV3 + CD1 + NH1	0.136	-0.078	-0.120	-0.211	-0.183	-0.117	-0.039	617
Sex + PV3 + CD3 + NH1 + CD3:PV3	0.101	-0.095	-0.126	-0.214	-0.188	-0.122	-0.038	618
Sex + PV3 + NH1	0.154	-0.069	-0.112	-0.217	-0.182	-0.115	-0.036	619
AY9 + Sex + PV3 + CD1 + AY9:PV3	0.082	-0.067	-0.119	-0.219	-0.197	-0.156	-0.052	620
AY9 + Sex + PV3 + CD1 + AY9:PV3 + CD1:PV3	0.081	-0.068	-0.118	-0.218	-0.198	-0.158	-0.050	621
AY5 + Sex + PV3 + CD1 + AY5:PV3	0.083	-0.070	-0.120	-0.219	-0.198	-0.156	-0.052	622
PV3 + CD1 + NH1 + CD1:PV3	0.133	-0.081	-0.123	-0.213	-0.189	-0.122	-0.037	623
AY5 + Sex + PV3 + CD1 + AY5:PV3 + CD1:PV3	0.082	-0.071	-0.120	-0.219	-0.200	-0.158	-0.050	624
AY9 + Sex + PV3 + AY9:PV3	0.130	-0.035	-0.099	-0.224	-0.202	-0.160	-0.053	625
AY5 + Sex + PV3 + AY5:PV3	0.131	-0.039	-0.100	-0.225	-0.205	-0.160	-0.054	626
AY9 + NH1	0.208	0.146	0.153	0.086	0.097	0.096	0.134	627
AY9 + NH5	0.172	0.122	0.150	0.110	0.116	0.103	0.150	628
AY5 + NH5	0.174	0.124	0.152	0.111	0.118	0.104	0.152	629
AY9 + NH3	0.184	0.129	0.153	0.107	0.115	0.103	0.146	630
Sex + PV3 + CD1 + NH1	0.124	-0.089	-0.131	-0.228	-0.194	-0.125	-0.044	631
AY5 + NH1	0.212	0.149	0.156	0.087	0.099	0.098	0.137	632
AY5 + NH3	0.187	0.131	0.155	0.108	0.116	0.104	0.148	633
Sex + PV3 + CD1 + NH1 + CD1:PV3	0.121	-0.093	-0.134	-0.231	-0.202	-0.130	-0.044	634
Sex + NH5	0.196	0.141	0.162	0.096	0.106	0.105	0.151	635
Sex + NH3	0.215	0.153	0.171	0.097	0.107	0.110	0.152	636
NH5	0.206	0.149	0.172	0.112	0.118	0.113	0.159	637
NH3	0.224	0.161	0.180	0.110	0.118	0.117	0.158	638
Sex + NH1	0.263	0.190	0.191	0.085	0.099	0.115	0.154	639
NH1	0.271	0.196	0.197	0.096	0.107	0.119	0.157	640
Sex	0.328	0.234	0.217	0.067	0.074	0.102	0.140	641
PV3 + CD5 + CD5:PV3	0.124	-0.129	-0.178	-0.301	-0.283	-0.215	-0.120	642
PV3 + CD5	0.127	-0.129	-0.179	-0.300	-0.281	-0.214	-0.120	643
PV3 + CD3	0.123	-0.137	-0.186	-0.308	-0.288	-0.215	-0.122	644
PV3 + CD3 + CD3:PV3	0.119	-0.137	-0.187	-0.308	-0.291	-0.217	-0.122	645
Sex + PV3 + CD5	0.116	-0.141	-0.192	-0.320	-0.295	-0.222	-0.127	646
Sex + PV3 + CD5 + CD5:PV3	0.113	-0.141	-0.192	-0.321	-0.298	-0.223	-0.127	647
Sex + PV3 + CD3	0.112	-0.148	-0.199	-0.327	-0.301	-0.223	-0.129	648
Sex + PV3 + CD3 + CD3:PV3	0.108	-0.149	-0.200	-0.327	-0.305	-0.225	-0.129	649
PV3 + CD1	0.141	-0.138	-0.206	-0.332	-0.307	-0.226	-0.134	650
PV3 + CD1 + CD1:PV3	0.138	-0.141	-0.208	-0.335	-0.313	-0.230	-0.135	651
Sex + PV3 + CD1	0.130	-0.149	-0.217	-0.349	-0.319	-0.233	-0.140	652
Sex + PV3 + CD1 + CD1:PV3	0.127	-0.152	-0.220	-0.352	-0.326	-0.238	-0.141	653
PV3	0.219	-0.120	-0.204	-0.365	-0.340	-0.251	-0.155	654
Sex + PV3	0.204	-0.132	-0.216	-0.382	-0.351	-0.256	-0.158	655

Supplementary Table S13: Number of influenza specimens by virus type, 2012/13 to 2019/20
Number and percentage of influenza specimens, collected and analysed as part of Finnish sentinel surveillance, by virus type and subtype/lineage.

Season	A(H1N1)	A(H3N2)	A, not subtyped	B/Victoria	B/Yamagata	B, not subtyped
2012/13[1]	165 (64%)	46 (18%)	6 (2%)			40 (16%)
2013/14[2]	67 (71%)	22 (23%)				6 (6%)
2014/15[3]	3 (2%)	97 (69%)		4 (3%)[b]	36 (26%)	
2015/16[4]	140 (79%)	12 (7%)		26 (15%)[b]	0 (0%)	
2016/17[5]	0 (0%)	131 (94%)[a]		0 (0%)	8 (6%)[b]	
2017/18[6]	6 (3%)	94 (41%)		7 (3%)	124 (54%)[b]	
2018/19[7]	73 (57%)	54 (42%)		0 (0%)	0 (0%)	
2019/20[8]	21 (70%)	8 (27%)		1 (3%)	0 (0%)	

[a] Antigenic mismatch between vaccine strain and circulating virus
[b] Lineage not included in trivalent vaccine composition

[1] Ikonen N, Murtopuro S, et al. Influenssakausi Suomessa, viikot 40/2012–20/2013: Seurantaraportti. Helsinki, Finland: National Institute for Health and Welfare, Finland, 2013.
[2] Ikonen N, Murtopuro S, et al. Influenssakausi Suomessa, viikot 40/2013–20/2014: Seurantaraportti. Helsinki, Finland: National Institute for Health and Welfare, Finland, 2014.
[3] Ikonen N, Murtopuro S, et al. Influenssakausi Suomessa, viikot 40/2014–20/2015: Seurantaraportti. Helsinki, Finland: National Institute for Health and Welfare, Finland, 2015.
[4] Ikonen N, Murtopuro S, et al. Influenssakausi Suomessa, viikot 40/2015–20/2016: Seurantaraportti. Helsinki, Finland: National Institute for Health and Welfare, Finland, 2016.
[5] Ikonen N, Murtopuro S, et al. Influenssakausi Suomessa, viikot 40/2016–20/2017: Seurantaraportti. Helsinki, Finland: National Institute for Health and Welfare, Finland, 2017.
[6] Ikonen N, Murtopuro S, et al. Influenssakausi Suomessa, viikot 40/2017–20/2018: Seurantaraportti. Helsinki, Finland: National Institute for Health and Welfare, Finland, 2018.
[7] Ikonen N, Haveri A, et al. Influenssakausi Suomessa, viikot 40/2018–20/2019: Seurantaraportti. Helsinki, Finland: National Institute for Health and Welfare, Finland, 2019.
[8] Ikonen N. Personal communication. 11 Feb 2020.

Supplementary Figure S1: Algorithm for assessing the absence/presence of residual confounding in a cohort study.

1. Identify basic elements of the study
- Study population, *e.g. elderly aged 65–100 years*
- Outcome of interest (Y), *e.g. laboratory-confirmed influenza*
- Exposure of interest (Z), *e.g. vaccination*

2. Choose negative control outcome (N) so that
- Z does not have a causal effect on N
- The set (X) of common causes of N and Z is identical to the set (X) of common causes of Y and Z

E.g. off-season hospitalization for acute respiratory infection

3. Check for confounding
In absence of other sources of bias such as measurement errors, the crude association between N and Z *is likely* to be confounded if the confidence interval of the crude hazard/risk ratio *does not include* the value of 1.

4. Identify potential confounders (X) based on subject matter knowledge, *e.g. age, sex, prior influenza vaccinations, comorbidities, health care utilization, medications, nursing home residency, socioeconomic status, smoking status*

5a. Define covariates (confounding variables) based on available data, *e.g. age* (X_1)*, sex* (X_2)*, previously vaccinated* (X_3)*, chronically diseased* (X_4)*, nights hospitalized* (X_5)

5b. Define covariate variants based on available data, *e.g.*
- X_{11}*: five age categories;* X_{12}*: nine age categories*
- X_{31}*: previous season;* X_{32}*: previous three seasons*
- X_{41}*: one-year;* X_{42}*: three-year;* X_{43}*: five-year history*
- X_{51}*: one-year;* X_{52}*: three-year;* X_{53}*: five-year history*

6a. Form sets of covariate variants and interaction terms, *e.g.* (X_{11}), (X_{12}), $(X_{11}; X_2)$, $(X_{12}; X_2)$, $(X_{11}; X_{31})$, $(X_{11}; X_{31}; I_{13})$, $(X_{11}; X_{32})$, $(X_{11}; X_{32}; I_{13})$, $(X_{12}; X_{31})$, $(X_{12}; X_{31}; I_{13})$, $(X_{12}; X_{32})$, $(X_{12}; X_{32}; I_{13})$, $(X_{11}; X_2; X_{31})$, $(X_{11}; X_2; X_{31}; I_{13})$, *etc.*, with I_{ij} marking the two-way interaction between X_i and X_j

6b. Rank sets of covariate variants and interaction terms according to the absolute value of the logarithm of the hazard/risk ratio adjusted for the respective covariates and interactions; if multiple comparable cohort studies can be conducted, *e.g. one for each season,* the sets can also be ranked by taking the sum of the absolute values over all studies

7. Adjust the hazard/risk ratio for the best set, i.e.
- The lower the rank the better
- The less complex the better

8. Check for residual confounding
In absence of other sources of bias such as measurement errors, residual confounding of the association between N and Z *is unlikely* if the confidence interval of the adjusted hazard/risk ratio *does include* the value of 1.

9. Conclusion
The absence of residual confounding of the covariate-adjusted association between N and Z suggests but does not prove absence of residual confounding of the corresponding, covariate-adjusted association between Y and Z.

Supplementary Figure S2: Influenza hazard ratio, 2012/13 to 2019/20 Ratio of the covariate-adjusted estimates of the hazard of laboratory-confirmed influenza in fully vaccinated study subjects and the corresponding hazard in the unvaccinated over time. Extreme estimates outside the plot region are depicted by triangles at $y = 2$. The gray shaded area highlights the month of the epidemic peak. The solid and the dashed lines show the point and 95% confidence interval estimates for the covariate-adjusted hazard ratio assuming proportional hazards.

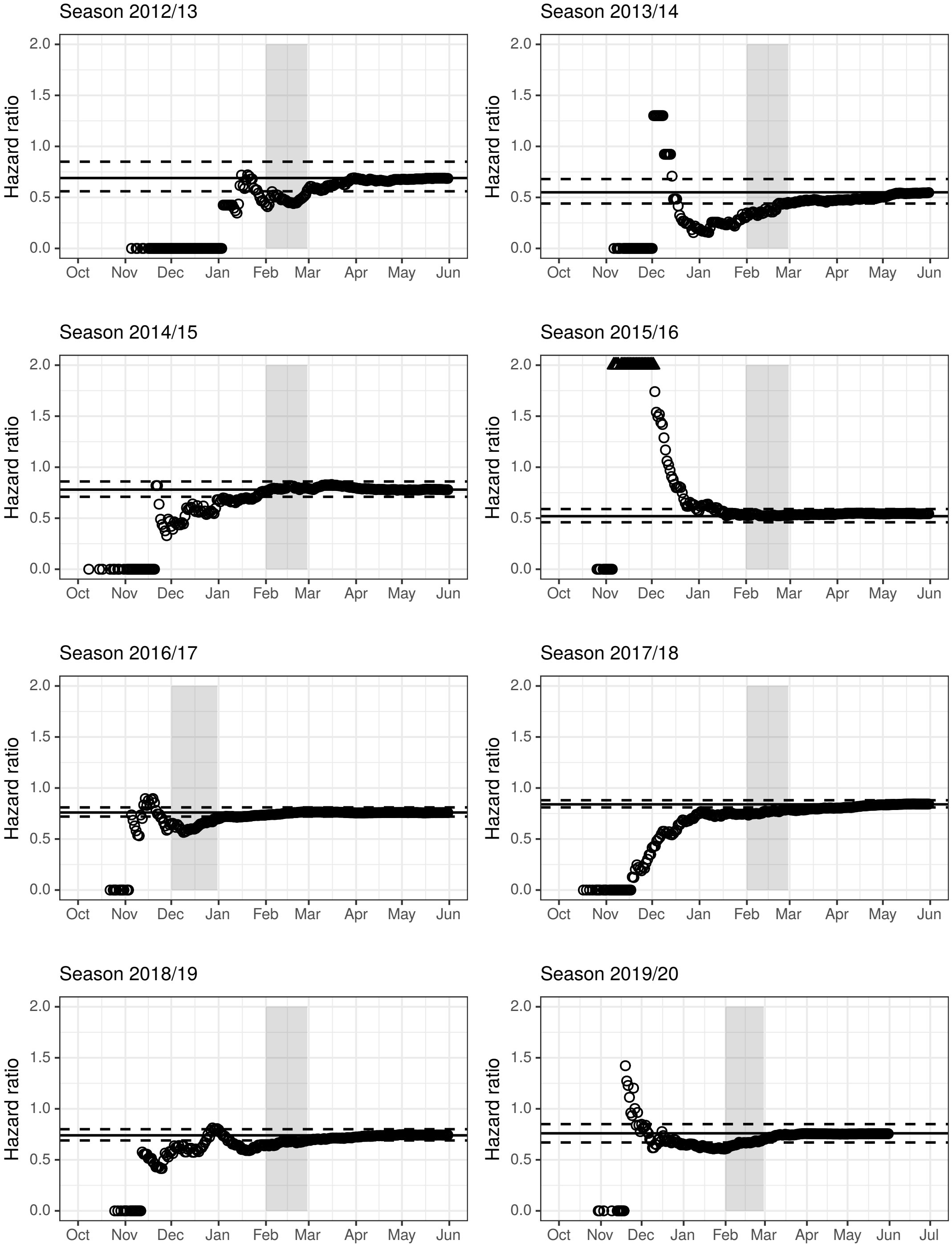

Supplementary Figure S3: Acute respiratory infection hazard ratio, 2012/13 to 2018/19 Ratio of the covariate-adjusted estimates of the hazard of hospitalization for acute respiratory infection in fully vaccinated study subjects and the corresponding hazard in the unvaccinated over time. Extreme estimates outside the plot region are depicted by triangles at $y = 5$.

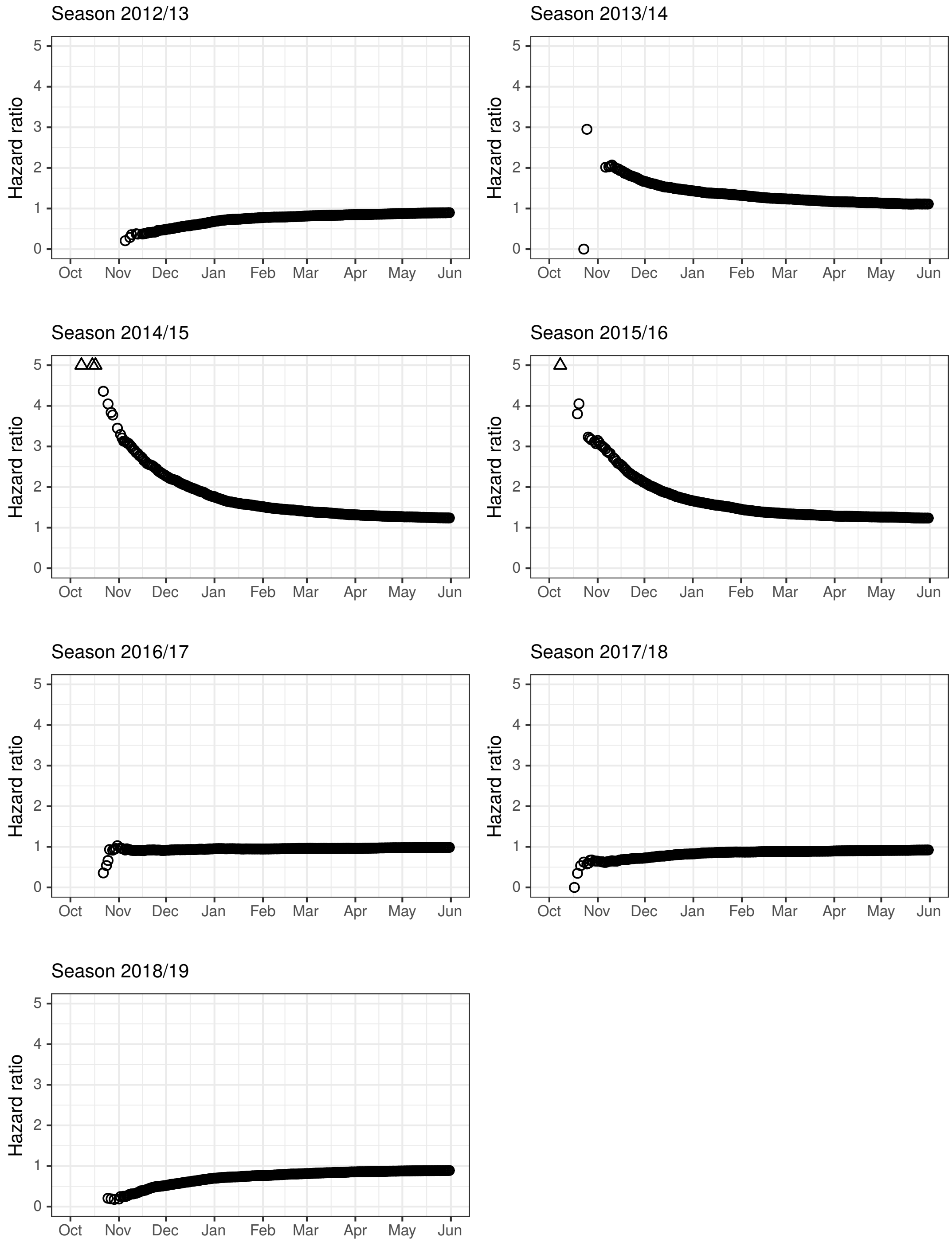

Supplementary Figure S4: Acute respiratory infection hazard ratio, 2013 to 2019 Ratio of the covariate-adjusted estimates of the hazard of hospitalization for acute respiratory infection in the vaccinated and the corresponding hazard in the unvaccinated over time using the original covariate definitions. The solid and the dashed lines show the point and 95% confidence interval estimates for the covariate-adjusted hazard ratio assuming proportional hazards.

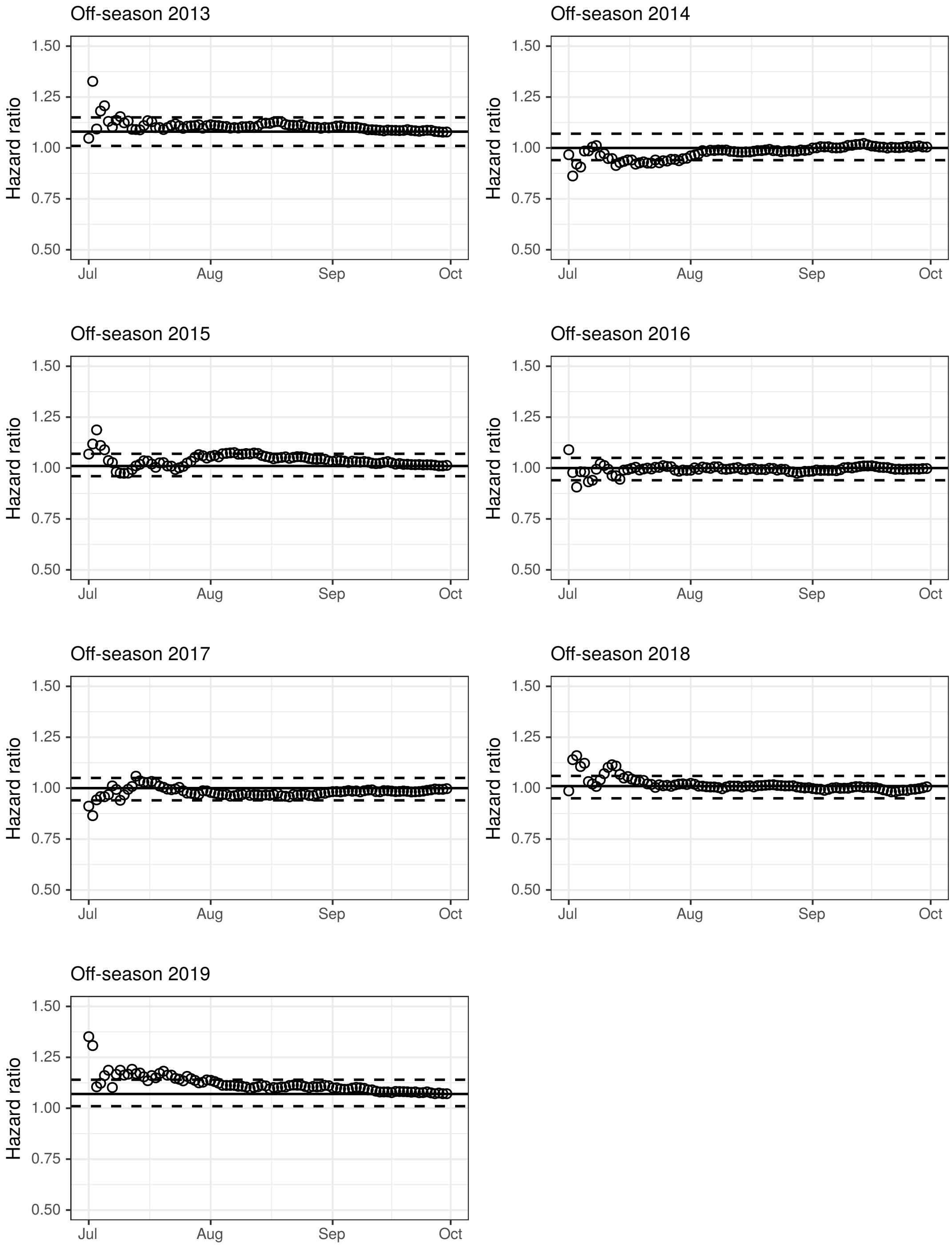

Supplement: Supplement [file 21-00054_BAUM_Supplement.pdf]
